# *In Silico* Driven Multi-Epitope Subunit Candidate Vaccine against Bovine Tuberculosis

**DOI:** 10.1155/2024/5534041

**Published:** 2024-09-04

**Authors:** Md. Atik Faysal, Fatema Yeasmin Tanni, Md. Mahfujur Rahman, Md Anisur Rahman, Md. Shahidur Rahman Chowdhury, Ho-Seong Cho, Md. Mukter Hossain, Md Bashir Uddin

**Affiliations:** ^1^Department of Medicine, Sylhet Agricultural University, Sylhet 3100, Bangladesh; ^2^School of Veterinary Medicine and Biomedical Sciences, University of Nebraska, Lincoln, NE 68583, USA; ^3^Department of Biochemistry, University of Nebraska-Lincoln, Beadle Center, 1901 Vine St, Lincoln, NE 68588, USA; ^4^College of Veterinary Medicine and Bio-Safety Research Institute, Jeonbuk National University, Iksan 54596, Republic of Korea; ^5^PMAC Veterinary Teaching Hospital, Sylhet Agricultural University, Sylhet 3100, Bangladesh

## Abstract

Bovine tuberculosis (bTB), caused by *Mycobacterium bovis*, poses significant zoonotic and economic challenges globally. The current prevention and treatment options are limited and increasingly complicated by the emergence of multidrug-resistant strains. This study employs reverse vaccinology and immunoinformatics to design a multi-epitope subunit vaccine targeting the MPB83, ArfA, DnaK, GrpE, and LpqH proteins of *M. bovis*. The T-cell and B-cell epitopes of the candidate vaccine were predicted and evaluated for antigenicity, allergenicity, and toxicity. The promising epitopes were then assembled into three vaccine constructs (bTBV1, bTBV2, and bTBV3) using appropriate adjuvants, pan HLA DR-binding epitope (PADRE), and linkers. The constructs were analyzed for physicochemical properties, 3D structure, cytokines induction and stability, followed by molecular docking with bovine CD molecules and toll-like receptor, TLR-9. Among the candidates, bTBV3 was chosen as one of the most promising vaccine candidates due to its high aliphatic index (67.60), lowest instability score (27.26), and a strong binding affinity. Molecular dynamics simulations and the results of interactions between the vaccine–receptor complexes (eigenvalue 2.318704e-06) show that the vaccine construct bTBV3 is stable. *In silico* immune simulation findings, such as elevated IgM levels and increased Th cell populations, suggest that the designed multi-epitope vaccine candidate bTBV3 elicits robust humoral and cellular immune responses, confirming the vaccine's potential efficacy. Additionally, codon optimization (CAI: 0.997 and GC: 54.687%) and *in silico* cloning facilitated efficient expression in *E. coli*. This study highlights the potential of bioinformatics-driven approaches in developing effective subunit vaccines against bTB, providing a foundation for experimental validation and future applications in combating this pervasive zoonotic disease, bovine tuberculosis.

## 1. Introduction

Bovine tuberculosis (bTB) is a major concern due to its widespread impact on livestock health, posing risks to both animals and humans. Studies in regions such as Gauteng, Bangladesh, and Nigeria have shown that the prevalence of bTB in cattle varies from 4.4% to 25.7% [1, 2, 3, 4]. The emergence of multidrug-resistant strains of *Mycobacterium bovis*, that causes bTB, has heightened the urgency for effective vaccines. In this context, researchers have been using immunoinformatics to design multi-epitope subunit vaccines against *Mycobacterium tuberculosis*, which is closely related to *M. bovis*. These *in silico*-driven approaches have led to the development of candidate vaccines with promising attributes [5, 6, 7, 8].

Currently, the treatment of bTB is relatively scarce, and antimicrobial combinations are frequently used. The problem of bovine tuberculosis treatment has become complicated and less effective when the infection is associated with multidrug and extensively drug-resistant *M. bovis*. The increasing number of treatment-resistant *Mycobacterium* TB strains are reported across the globe suggesting that drug discovery cannot be the primary means of eradication [9]. In this case, the only TB vaccine available is a bacillus Calmette-Guérin (BCG) made from an attenuated *M. bovis* strain. The vaccine's effectiveness, on the other hand, ranges from 0% to 80% in various populations, with relatively poor effectiveness in many tropical areas where the vaccine is most required [10].

In recent years, multi-epitope vaccines have attracted much attention due to their higher immunity and lower allergenicity advantages than conventional vaccines. Our study on *in silico* bTB vaccine development contributes significantly to the field by utilizing computational techniques to predict epitopes for TB proteins, design multi-epitope subunit vaccines efficiently. By employing *in silico* techniques like epitope prediction, molecular docking, and immune simulations, researchers can identify potential antigens and develop multi-epitope vaccines with high immunogenicity and safety profiles [5, 6, 7, 8]. This approach accelerates the process of vaccine design by targeting specific proteins of *M. tuberculosis*, ensuring broad population coverage and enhanced efficacy against multidrug resistant strains. Furthermore, *in silico* tools enable the assessment of vaccine stability, immune response activation, and potential interactions with host receptors, paving the way for the development of novel bTB vaccines that could address the challenges posed by the current BCG vaccine [11, 12] and contribute to the global eradication of tuberculosis.

This study introduces a groundbreaking approach in the field of bTB vaccination. Here, we approach *in silico* techniques to design a multi-epitope subunit candidate vaccine targeting the MPB83, ArfA, DnaK, GrpE, and LpqH proteins of *M. bovis*. We have leveraged reverse vaccinology and immunoinformatics to predict B-cell, helper T-cell, and cytotoxic T-cell epitopes crucial for inducing a robust immune response [5, 6, 7, 8]. We explored the physicochemical properties and receptor–binding interaction of the multi-epitope vaccine followed by molecular dynamics simulation of the vaccine–receptor complexes. By integrating these epitopes into a single vaccine construct, we aim to create a safe, potent, and tailored vaccine against bTB. Our innovative approach not only enhances the understanding of bTB vaccination but also sets a precedent for the development of effective vaccines against complex pathogens by harnessing computational tools for epitope prediction and vaccine design.

## 2. Materials and Methods

**Figure 1:**
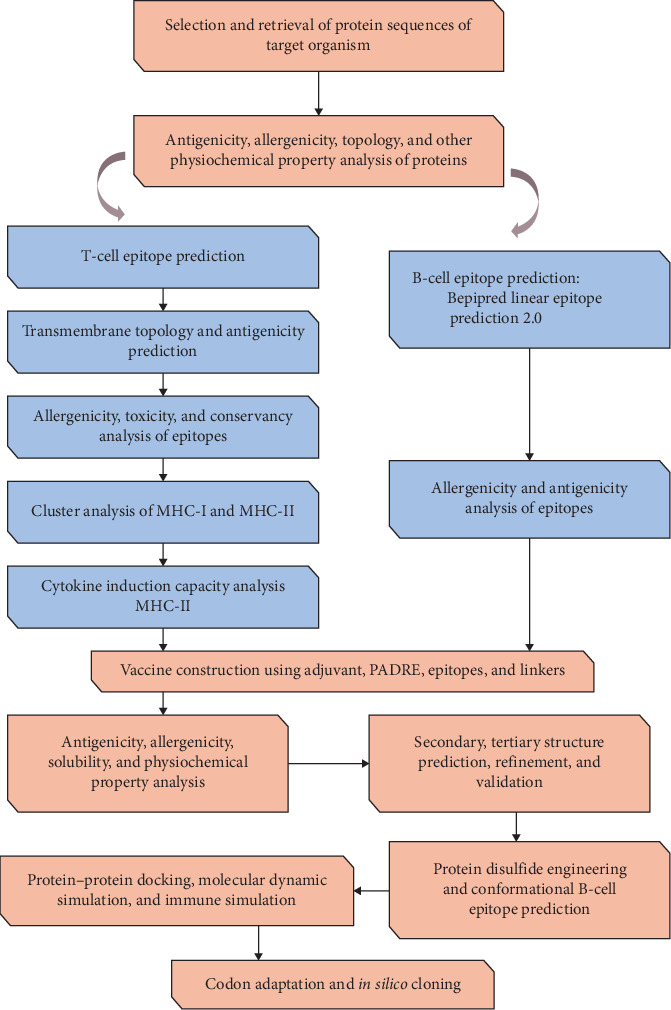
Flowchart depicting the steps required in the *in silico* design of a multi-epitope candidate vaccine against bTB. The process encompasses numerous key phases, including retrieval of target protein sequence, epitope prediction, construction of vaccine candidates, physicochemical properties, 3D model prediction, MD simulation, molecular docking to the immune receptor, and *in silico* cloning.

In this study, an *in silico* reverse vaccinology method was utilized to generate vaccine candidates for bTB. The consecutive steps used in this study to develop and evaluate a multi-epitope candidate vaccine against bTB are depicted in Figure 1.

### 2.1. Target Protein Sequences Selection and Retrieval

The NCBI database (https://www.ncbi.nlm.nih.gov/) was employed to conduct thorough research and choose potential proteins for this study [13]. A total of five proteins were acquired from the UniProt dataset (https://www.uniprot.org/): cell surface glycolipoprotein MPB83, peptidoglycan-binding protein ArfA, chaperone protein DnaK, protein GrpE, and lipoprotein LpqH [14].

### 2.2. T-Cell Epitope Prediction

The IEDB server's T-cell epitope prediction website (http://tools.iedb.org/main/tcell/) has been utilized to anticipate T-cell epitopes (MHC-I and MHC-II) by relying on common regions. The MHC class I restricted CD8^+^ cytotoxic T-lymphocyte (CTL) epitopes were obtained using the NetMHCpan EL 4.1 prediction method [15]. The alleles BoLA-3 : 00101, BoLA-3 : 00201, BoLA-5 : 00301, BoLA-3 : 00401, BoLA-2 : 00501, BoLA-2 : 00601, BoLA-2 : 00801, BoLA-1 : 00901, and BoLA-3 : 01001 were considered, with a sequence length of 9. The MHC class II restricted CD4^+^ helper T-lymphocyte (HTL) epitopes were acquired for HLA-DRB1*⁣*^*∗*^03 : 01, HLA-DRB1*⁣*^*∗*^0401, HLA-DRB1*⁣*^*∗*^0801, HLA-DRB1*⁣*^*∗*^1101, HLA-DRB1*⁣*^*∗*^1301, HLA-DRB1*⁣*^*∗*^1401 alleles, while maintaining a sequence length of 12 using the Sturnilo method [16, 17].

### 2.3. Transmembrane Topology, Antigenicity, Allergenicity, Conservancy, and Toxicity Screening of T-Cell Epitopes

The assessment of antigenicity for the epitopes (with a threshold value of 0.50) was conducted using the VaxiJen v2.0 server (http://www.ddg-pharmfac.net/vaxijen/VaxiJen/VaxiJen.html) [18]. The transmembrane topology of the epitopes was predicted using the online programs TMHMM (http://www.cbs.dtu.dk/services/TMHMM/) and HMMTOP (http://www.enzim.hu/hmmtop/index.php). The antigenic epitopes with the highest potency were chosen for further investigation. In order to forecast the selected epitope allergenicity, online resources, namely, AllerTOP v2.0 (~94% accuracy, https://www.ddg-pharmfac.net/AllerTOP/) and AllergenFP v1.0 (~88% accuracy, https://ddg-pharmfac.net/AllergenFP/), were employed [19, 20]. The epitope conservancy analysis tool on the IEDB server (http://tools.iedb.org/conservancy/) was used to identify highly conserved epitopes (with a threshold value of >60%) against multiple *Mycobacterium* protein sequences [21]. The blast against *M. bovis* (taxid:1765), *M. bovis* BCG (taxid:33892), *M. bovis* subsp. *caprae* (taxid:115862), *M. bovis* AF2122/97 (taxid:233413), and *M. bovis* BCG str. Korea 1168P (taxid:1206780) was conducted using the NCBI server's BlastP tool [22, 23]. A support vector (SVM-Swiss-Prot) was utilized to assess toxicity using the ToxinPred program (Matthews correlation coefficient (MCC) is 0.91) (http://crdd.osdd.net/raghava/toxinpred/) [24].

### 2.4. Cluster and Cytokine Induction Capacity Analysis of Selected MHC-II Epitopes

MHC cluster 2.0 (http://www.cbs.dtu.dk/services/MHCcluster/) was employed to examine the clustering patterns of MHC alleles using the NetMHCpan-2.8 prediction technique [25]. The study used 50,000 peptides and 100 bootstrap replicates. A total of nine members from the BoLA (MHC class I) and six representatives from the HLA-DR (MHC class II) were chosen.

The generation of cytokines is restricted to helper T-cells, thus the induction of cytokines such as IFN-gamma and IL-4 was predicted using MHC-II epitopes. To accomplish this, the prediction servers IFNepitope (https://webs.iiitd.edu.in/raghava/ifnepitope/design.php) and IL4pred (https://webs.iiitd.edu.in/raghava/il4pred/design.php) were utilized [26, 27].

### 2.5. B-Cell Epitope Prediction, Antigenicity, and Allergenicity Analysis

The B-cell lymphocytic (BCL) epitopes were chosen by the IEDB server (http://tools.iedb.org/bcell/) depending on their sequence length (sequences longer than 10 and shorter than 40 amino acids). The Bepipred Linear Epitope Prediction 2.0 technique [28] was employed to predict these epitopes. The epitopes' antigenicity was determined using the VaxiJen v2.0 server [18]. In order to forecast the allergenicity of the chosen epitopes, two online tools, namely, AllerTOP v2.0 and AllergenFP v1.0, were utilized [19, 20].

### 2.6. Multi-Epitope Vaccine Construction

The best CTL, HTL, and BCL epitopes were combined to develop a vaccine construct bTBV1, bTBV2, and bTBV3 against bovine tuberculosis. Three distinct adjuvant sequences were used in the development of the vaccine: beta-defensin-3 (AAV41025.1), HABA protein (*M. tuberculosis*, AGV15513.1), and 50S ribosomal protein L7/L12. All vaccines were created in the following order: PADRE sequence, adjuvant, CTL, HTL, and BCL epitopes. Various linkers such as EAAAK, GPGPG, GGGS, and KK were employed to facilitate the connection of all sequences [29, 30].

### 2.7. Allergenicity, Antigenicity, Topology, Solubility, and Physicochemical Property Analysis of the Primary Sequences and Constructed Vaccines

The allergenicity of the primary protein sequences and vaccine construction was assessed using the Internet services AllergenFP v1.0 and AllerTOP v2.0. The antigenicity of both cases was determined using the VaxiJen v2.0 server. The TMHMM Server version 2.0 (http://www.cbs.dtu.dk/services/TMHMM/) [31] and HMMTOP server (http://www.enzim.hu/hmmtop/index.php) [32] were employed to determine the protein sequence topology. Protein–sol (https://protein-sol.manchester.ac.uk/) was employed to ascertain the vaccines' solubility [33]. The ExPASy service's tool, ProtParam, was employed to effectively characterize the physicochemical properties of protein sequences and vaccine candidates [34].

### 2.8. Prediction of 3D Structure, Refinement, and Validation of the Vaccine Proteins

The online program PRISPRED (http://bioinf.cs.ucl.ac.uk/psipred/) [35] was utilized to forecast the proportions of alpha-helix, beta-sheet, and coil structures in the constructs. The tertiary structures of the vaccines were generated utilizing the distance-based technique offered by the online platform RaptorX (http://raptorx.uchicago.edu/) [36]. Discovery Studio Visualizer was employed to visualize the three-dimensional structure.

The 3D structure was modified utilizing GalaxyRefine (http://galaxy.seoklab.org/) [37]. The validity of the vaccine proteins was subsequently assessed using the PROCHECK (https://servicesn.mbi.ucla.edu/PROCHECK/) [38].

### 2.9. Disulfide Engineering and Conformational B-Cell Epitope Prediction of the Vaccine

The disulfide engineering of the bTBV3 vaccine protein was conducted using the online program Disulfide by Design 2 v12.2 (http://cptweb.cpt.wayne.edu/DbD2/). A structural model was employed to find residue pairs in proteins that can be modified into cysteines for the formation of the novel linkage. It was done in the context of the creation of novel disulfide bonds within proteins [39].

Ellipro (http://tools.iedb.org/ellipro/) tool was utilized to anticipate the discontinuous epitopes within the bTBV3 vaccine [40]. In addition, Jmol [41] was used to facilitate the visualization of the protein's linear and noncontiguous epitopes in a three-dimensional format.

### 2.10. Protein–Protein Docking and Molecular Dynamics Simulation

The docking of different bovine CD molecules and TLR-9 with the vaccine construct (bTBV3) was performed. In the initial stage, we conducted a search in the RCSB protein data bank (https://www.rcsb.org/) to retrieve the three-dimensional conformation of receptor molecules [42]. Subsequently, the PatchDock (https://bioinfo3d.cs.tau.ac.il/PatchDock/php.php) server was employed with its default settings to determine the most favorable positions and orientations [43]. Consequently, the top 10 solutions were uploaded to the FireDock web server (https://bioinfo3d.cs.tau.ac.il/FireDock/php.php) to enhance the interaction between protein receptor molecules [44]. The option that consumed the least global energy was then selected. The HDOCK algorithm was employed to perform molecular docking of the ligand to the Toll-like receptor 9 (TLR-9) (http://hdock.phys.hust.edu.cn/) [45].

The molecular dynamics simulation for the bTBV3 vaccine was performed using the iMODS server (http://imods.chaconlab.org/) [46]. Furthermore, the ligand–receptor complex obtained from the HDOCK server was employed in the molecular dynamics simulation.

### 2.11. Immune Simulation

An *in silico* immune response was generated using the C-IMMSIM immune server (http://www.cbs.dtu.dk/services/C-ImmSim-10.0/) [47]. The simulation volume and steps were set to 1050, with no LPS in the vaccine injection. The simulation time step in real life is about a few hours, and the simulation was executed with the default settings. The injections were administered in the following order: Ag1, Ag2, and Ag3; with a 4-week interval between each administration. The C-ImmSim model simulates the humoral and cellular responses of the mammalian immune system to a vaccine design [48].

### 2.12. Codon Adaptation and *In Silico* Cloning

The bTBV3 vaccine underwent codon adaptation utilizing the JCat server (http://www.jcat.de/) [49]. The *E. coli* strain K12 was chosen as the host. We excluded the Rho-independent transcription termination site, the prokaryote ribosome binding site, and the *EcoR1* and *BglII* restriction enzyme cleavage sites. To validate the efficacy of restriction cloning, the vaccine protein bTBV3 was subjected to sequence inversion, followed by conjugation with EcoR1 and BglII restriction sites located at the N-terminal and C-terminal regions, respectively. Finally, the SnapGene restriction cloning module (https://www.snapgene.com/) was employed to facilitate the insertion of the modified DNA sequences into the restriction *EcoR1* and *BglII* sites of the pET-28a(+) vector [50, 51].

## 3. Results

### 3.1. Selection of Proteins

The possible five proteins were selected as potential vaccine candidates from the NCBI's database. The target proteins were cell surface glycolipoprotein MPB83 (accession no—P0CAX7), peptidoglycan-binding protein ArfA (accession no—A1KH31), chaperone protein DnaK (accession no—P0A5C0), protein GrpE (accession no—A1KFH3), and lipoprotein LpqH (accession no—A0A0H3M9Z0).  Table 1 represents the list of the amino acid sequences of those proteins.

### 3.2. Antigenicity, Allergenicity, Topology, and Physicochemical Analysis

The peptides selected from the protein sequences were found to be potentially antigenic, as their prediction scores exceeded the predefined threshold value of 0.50. This suggests that these proteins possess the ability to elicit an immune response in the host, potentially leading to the development of protective immunity. Moreover, the extracellular localization of specific proteins' transmembrane structures was observed. It was revealed that all proteins exhibited nonallergenic properties. All proteins exhibited a decreased instability score and an increased aliphatic index. *Supplementary 1* contains the antigenicity scores and other physicochemical analyses of the chosen proteins.

### 3.3. T-Cell Epitope Prediction and Determination of Transmembrane Topology, Antigenicity, Allergenicity, Conservancy, and Toxicity of the Epitopes

The T-cell epitopes (MHC class I and MHC class II) of the five proteins were determined using the IEDB server. In addition, the top epitopes for allergenicity, toxicity, and conservancy tests were chosen based on antigenicity scores (AS) and transmembrane topology (*Supplementary 1*).

The results of the allergenicity, toxicity, and conservancy investigations indicate that the epitopes exhibit nonallergenic and nontoxic properties, with a conservancy of about 83%–100%. Consequently, epitopes that exhibited the highest antigenicity score and fulfilled further criteria were chosen for the development of a vaccine against bTB (Table 2).

### 3.4. Cluster Analysis and Cytokine Induction Capacity Assessment of MHC-II Epitopes

**Figure 2:**
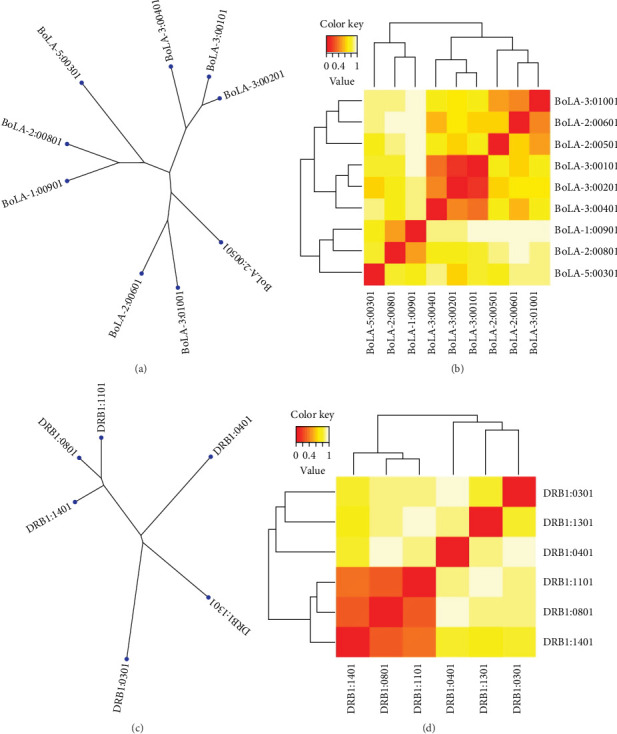
Cluster analysis and phylogenetic tree: (a, b) MHC-I and (c, d) MHC-II alleles expression. Heatmap of correlation coefficients among MHC-I and MHC-II alleles' attributes. Red regions indicate robust interactions and yellow regions indicate milder interactions.

MHC cluster 2.0 was used to evaluate potential interactions between the expected epitopes and MHC class I and MHC class II alleles. The algorithm provides groups of alleles in phylogenetic order. The server displayed the findings as an MHC specificity tree and a heat map. The findings of the study are depicted in Figure 2, whereby red areas represent strong interactions and yellow areas indicate weaker interactions.

Based on the findings of the study on the cytokine induction capacity of MHC-II epitopes, it was observed that 20% and 70% of the epitopes exhibited the ability to elicit interferon-gamma (IFN-*γ*) and interleukin-4 (IL-4) production, respectively (Table 3). T-helper 1 (Th1) cells are responsible for the production of interferon and the activation of macrophages, both of which play crucial roles in the elimination of pathogens within host cells. Thus, IFN-*γ* assists the immune system in combating bacterial, viral, and tumor development by modulating the immune system [26]. On the other hand, IL-4 secretion is required for antibody isotype shifting and promotes IgE synthesis. Additionally, this cytokine stimulates the multiplication and specialization of antigen-presenting cells [27].

### 3.5. B-Cell Epitope Prediction and Allergenicity, Antigenicity Analysis

The B-cell epitopes of all proteins in this study were anticipated utilizing the IEDB's Bepipred technique. The epitopes that we have chosen demonstrated higher antigenicity and a lack of allergenicity. Therefore, it is expected that the suggested vaccine architecture would confer broad-spectrum protection to the host. The potential B-cell epitopes are listed in Table 4.

### 3.6. Construction of Vaccine

Three candidate vaccine constructs have been generated (i.e., bTBV1, bTBV2, and bTBV3). Every construct consisted of a protein adjuvant and a PADRE peptide sequence [29], while the remaining portion was filled with T- and B-cell epitopes together with their respective linkers. The vaccines were formulated by employing GGGS, GPGPG, and KK linkers to facilitate the fusion of 20 T-cell epitopes, consisting of 10 MHC-I and 10 MHC-II epitopes, together with 10 B-cell epitopes. Consequently, the three vaccines were identical except for the adjuvant sequences, and all three had the same epitopes. The constructions of bTBV1, bTBV2, and bTBV3 consisted of 516, 676, and 601 residues, respectively (Table 5).

### 3.7. Allergenicity, Antigenicity, Solubility Prediction, and Physicochemical Characterization of the Vaccine Constructs

**Figure 3:**
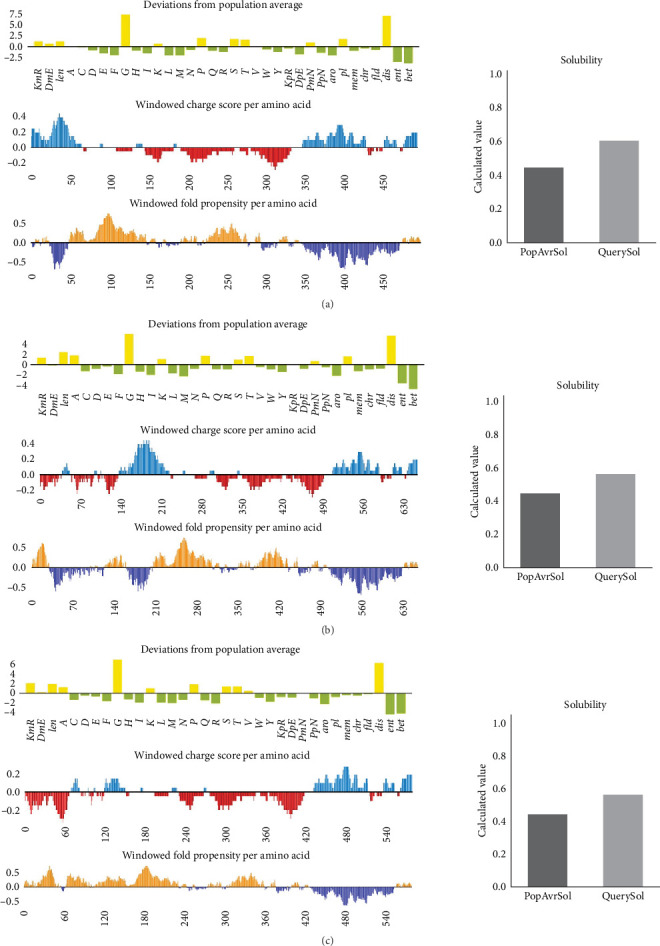
Solubility analysis of the vaccine constructs. The solubility of all three-vaccine construct was shown to exceed the 0.45 cutoff value: (a) bTBV1, (b) bTBV2, and (c) bTBV3.

The tools Allertop v2.0 and AllergenFP were employed to forecast the nonallergenic properties of vaccine formulations. All three vaccines yielded negative results in allergenicity tests and were subsequently verified to exhibit antigenicity. The solubility of all three structures surpassed the threshold value of 0.45 (Table 6 and Figure 3). The vaccines possess molecular weights of 49.43, 66.44, and 57.71 kDa, respectively. The half-life of the proposed structure in *E. coli* was estimated to be more than 10 hr. The aliphatic index and GRAVY value of bTBV3 were determined to be 67.60 and −0.298, respectively. These values suggest that the protein exhibits a high degree of thermostability and hydrophilicity. The bTBV3 had an instability score of 27.26, signifying its status and level of stability. Thus, the constructed protein (bTBV3) was classified as stable and it was determined that the construct possessed the requisite attributes to induce an immunogenic reaction within the organism (Table 7).

### 3.8. Secondary and Tertiary Structure Prediction of the Constructed Vaccines

According to secondary structure analysis, the bTBV1 vaccine had the fewest amino acids (14.15%) in the alpha helix formation and the most amino acids. Meanwhile, the bTBV2 and bTBV3 constructs have an alpha-helix of 30.03% and 21.63%, respectively (*Supplementary 1 and Supplementary 2*).

The RaptorX website's distance-based structure prediction algorithm provided five tertiary structure models for each vaccine during the structure prediction process. The models are ranked based on the anticipated root mean square deviation (RMSD) of the three-dimensional model, as determined by the experimental design. There is a positive correlation between the size of an object and the likelihood of it, including high-quality 3D models [52].

### 3.9. Refinement and Validation of the Tertiary Modeled Structure

**Figure 4:**
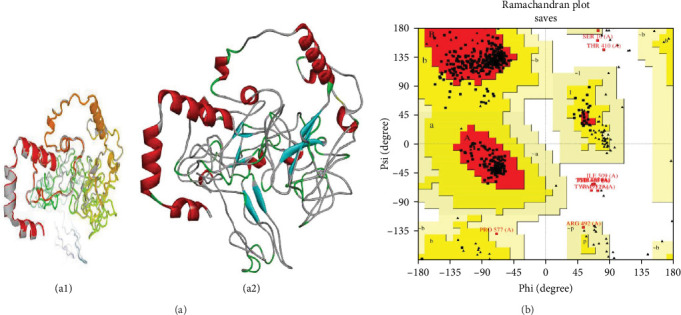
3D structure refinement and validation of bTBV3: (a) refined structure: (a1) shows the crystal structure, and in (a2), the backbone of bTBV3 is shown with red ribbons and cyan arrows to indicate helices and strands, respectively, and (b) Ramachandran plot. The Ramachandran plot analysis shows that in the initial model, 88.7% of residues are found highly preferred for bTBV3 construct.

The GalaxyRefine algorithm enhanced the accuracy of three-dimensional protein structures by generating a set of five refined models for each vaccine construct. The structure that exhibited the highest level of refinement (Figure 4(a)) was subsequently selected. After refinement by Ramachandran plot analysis, 88.7% of residues in vaccine bTBV3 were located inside the most favored region. Additionally, 9% of residues were found in the additional authorized regions, while 0.5% were situated in the liberally allowed regions. Furthermore, 1.9% of residues were identified as prohibited regions (Figure 4(b) and *Supplementary 2*). *Supplementary 1* contains the validation scores for bTBV1, bTBV2, and bTBV3 vaccine constructs. It is expected that superior sound quality will surpass higher levels of residue [38]. Subsequently, bTBV3 emerged as the optimal choice for a potential vaccine candidate due to its abundance of residues in the most desirable region, its elevated aliphatic index, and its minimal instability index.

### 3.10. Protein Disulfide Engineering and Conformational B-Cell Epitope Prediction

**Figure 5:**
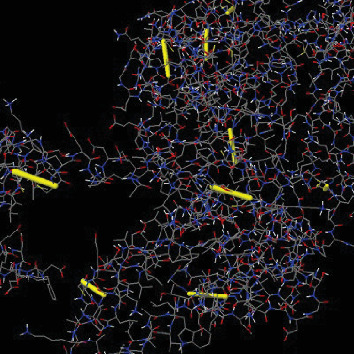
The disulfide engineering of the multi-epitope vaccine constructs bTBV3's 3D structure (mutant form disulfide bonds are shown by yellow sticks).

The DbD2 server identified 76 pairs of amino acid residues within bTBV3 as capable of forming disulfide bonds. After calculating the energy of the residue pairs, only nine pairs satisfied the necessary conditions for disulfide bond formation. The residue pairs identified include GLU 111–ALA 114, GLY 129–PRO 176, ALA 200–SER 222, GLY 247–THR 263, VAL 310–ALA 329, ALA 379–GLY 386, GLY 431–GLN 456, GLY 433–TYR 444, and GLN 517–SER 581 (Figure 5 and *Supplementary 1*). The energy threshold employed for residue filtration was below 2.20. Cysteine substitutions were performed on all 18 residues.

**Figure 6:**
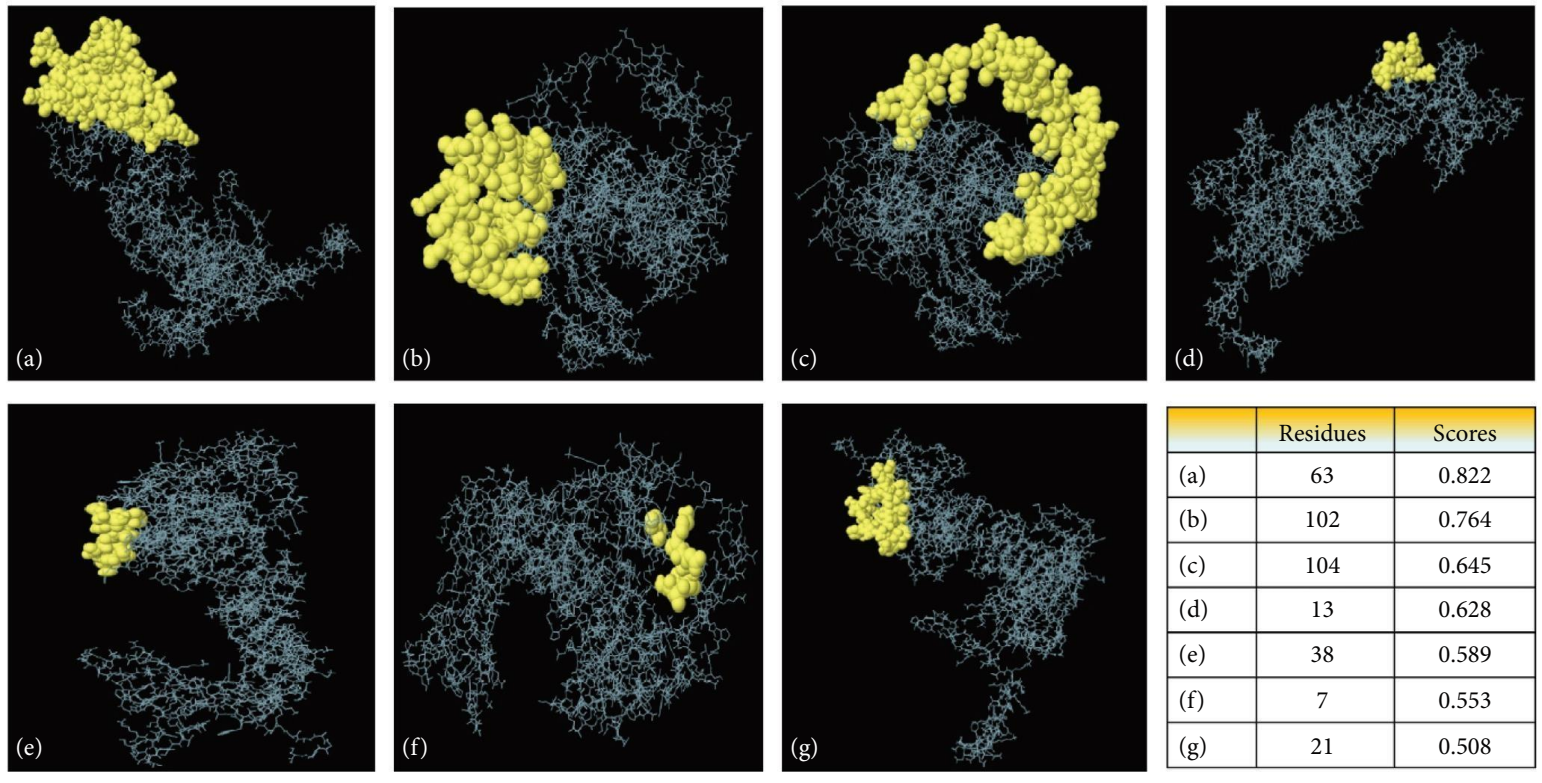
The discontinuous B-cell epitopes in the multi-epitope vaccine 3D model. The vaccine construct is depicted by gray sticks, while the discontinuous B-cell epitopes are shown by yellowish spheres: (a) 63 residues with a score of 0.822; (b) 102 residues with a score of 0.764; (c) 104 residues with a score of 0.645; (d) 13 residues with a score of 0.628; (e) 38 residues with a score of 0.589; (f) seven residues with a score of 0.553; and (g) 21 residues with a score of 0.508.

Based on the improved three-dimensional structure of bTBV3, the Ellipro server predicted conformational B-cell epitopes. A total of seven distinct epitope groups were identified, with projected values ranging from 0.508 to 0.822 for their corresponding residues (Figure 6 and *Supplementary 1*).

### 3.11. Protein–Protein Docking and Molecular Dynamics Simulation

**Figure 7:**
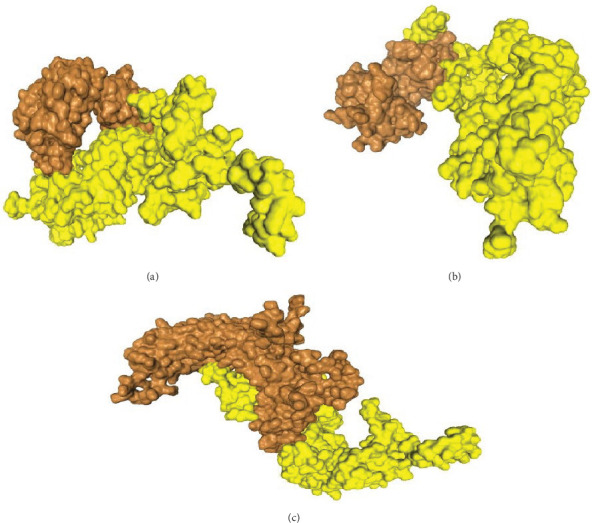
Molecular docking structure of bTBV3 vaccine construct by HDOCK with (a) BoLA-A11, (b) CD8, and (c) TLR-9.

The server identified the complex structure by analyzing the complementary score, the atomic contact energy (ACE), and the compound's estimated interface area (Table 8). The docking process between bTBV3 and TLR-9 was subsequently conducted via the HDOCK server (Figure 7). The observed negative scores indicate a robust interaction between the vaccine construct bTBV3 and the ligand TLR-9.

**Figure 8:**
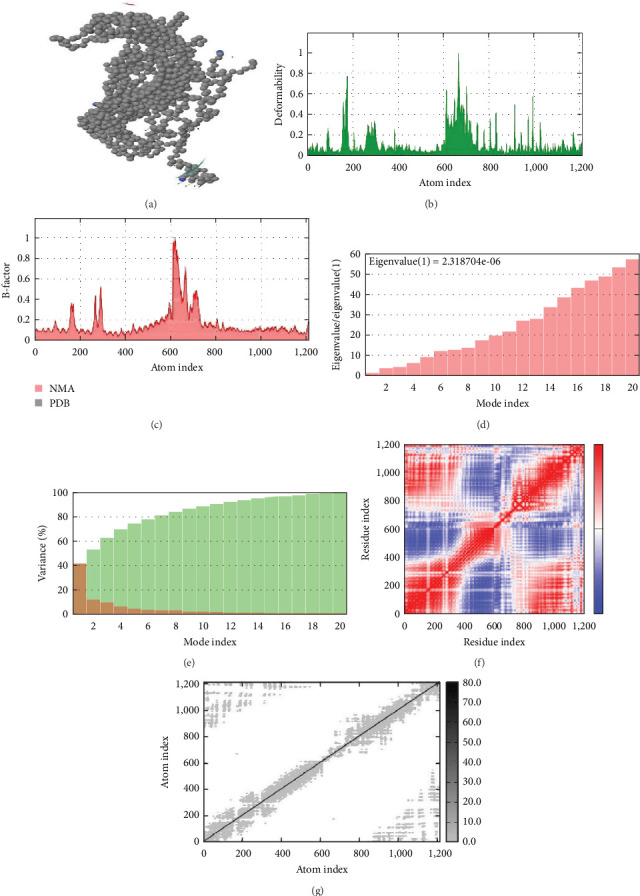
Molecular dynamics simulation of vaccine construct bTBV3. Stability of vaccine construct was analyzed by (a) NMA mobility, (b) deformability, (c) B-factor, (d) eigenvalues, (e) variance, (f) covariance map, and (g) elastic network analysis.

The stability of the vaccine construct bTBV3 was investigated using the mobility analysis, B-factor, eigenvalues, deformability analysis, covariance map, and the suggested elastic network model (Figure 8). The results of the study revealed that the specific placement of the hinge inside the chain did not have a significant impact. Additionally, the B-factor column provided an average of the root mean square error. Furthermore, the projected elevated eigenvalue of 2.318704e-06 provides evidence that the deformation of the bTBV3 vaccine protein is improbable.

### 3.12. Immune Simulation

**Figure 9:**
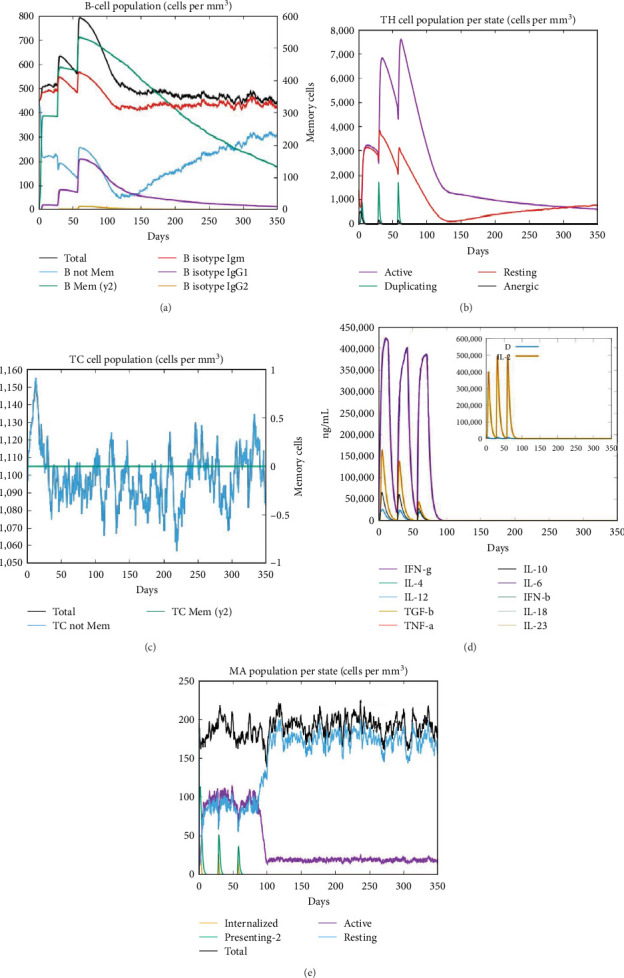
Immune response after administering vaccine construct bTBV3: (a) B-lymphocytes cell population; (b) CD4^+^ helper T-cells population per state; (c) CD8^+^ T-cytotoxic lymphocytes count; (d) induced levels of the cytokine and Simpson index, D; and (e) number of macrophages per cubic millimeter can present on both MHC class I and class II molecules antigenic peptides.

The immune simulation findings of the ImmSim server show concurrence with the reported immunological responses. The levels of IgM were found to be high, suggesting the occurrence of the primary immune response. Furthermore, an elevation in the B-cell populace led to an augmentation in the expression of immunoglobulins, resulting in a reduction in antigen concentration. Moreover, the development of memory is linked to a gradual increase in the number of Th (helper) cells. The findings of this study demonstrated that the T-cell population exhibited a high level of sensitivity during the process of memory formation, whereas the populations of other immune cells remained consistent (Figure 9).

### 3.13. Codon Optimization and *In Silico* Cloning of the Multi-Epitope Vaccine Candidate

**Figure 10:**
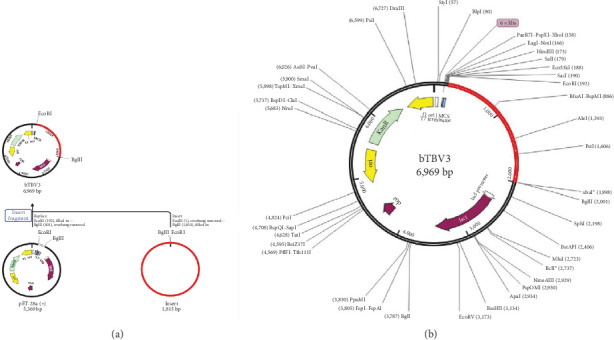
*In silico* cloning of bTBV3 vaccine candidate. The gene coding DNA sequence of the multi-epitope vaccine (shown in red) surrounded between *EcoRI* (192) and *Bg1II* (2001) into the pET-28a (+) expression vector (shown in black): (a) transition of cloning process and (b) bTBV3 cloned DNA in vector.

The CAI and GC content of the vaccine candidate bTBV3 were found to be 0.997% and 54.687%, respectively (*Supplementary 2*). A DNA fragment with a length of 1,803 base pairs was generated, intentionally lacking the EcoR1 and BglII restriction sites, enabling the process of cloning. The codons were inserted into the pET28a (+) vector, which had two restriction enzyme sites (EcoR1 and BglII), resulting in the production of a recombinant plasmid with a total length of 6,969 base pairs (Figure 10).

## 4. Discussion

Prophylactic vaccination is the predominant strategy for mitigating the transmission of tuberculosis. The only vaccine approved for administration in cattle as a preventive measure against bovine tuberculosis is Bacille Calmette–Guérin (BCG). In underdeveloped nations, tuberculosis is common in both domestic and wild animals. So, finding safe and effective vaccinations against bTB remains a critical challenge. A novel type of vaccine candidate that might address the aforementioned problems is the multi-epitope vaccine. This particular form of vaccine is composed of a recombinant protein made up of several or overlapping epitopes (peptides).

Here, reverse vaccinology methods with various bioinformatics tools were used to develop promising vaccine candidates against bTB. The identification of potential vaccine targets among bacterial proteins was conducted by a search of the NCBI database. These include the cell surface glycolipoprotein MPB83, the peptidoglycan-binding protein ArfA, the chaperone protein DnaK, the protein GrpE, and the lipoprotein LpqH. All proteins possess the inherent capacity to contribute to the organism's survival. MPB83 helps to induce TLR1/TLR2-dependent expression of human (host) matrix metalloproteinase-9 (MMP9) [53]. ArfA is an operon-encoded membrane protein that is required for bacterial growth in acidic environments. Its roles in acid stress resistance and peptidoglycan binding imply a link between the acid stress response and the physicochemical properties of the mycobacterial cell wall [54]. The protein DnaK, which is found in prokaryotes, induces both innate and adaptive immune responses [55]. GrpE also interacts with dendritic cells to elicit a partial immune response from T1-type T-cells [56]. Thus, DnaK and GrpE are both required for the survival of *M. tuberculosis*, rendering them highly promising candidates for the development of therapeutic interventions against tuberculosis. LpqH is a 19 kDa mycobacterial lipoprotein that stimulates T-cell proliferation and induces apoptosis in macrophages via a caspase-dependent/independent loss of mitochondrial transmembrane potential [57]. The sequences were retrieved from the UniProt database (Table 1). The proteins' antigenicity, allergenicity, and topology studies predicted that they were potentially antigenic, nonallergenic, and had an outer topology (*Supplementary 1)*. Based on these promising findings, it was postulated that the selected epitopes from the protein sequences were the most suitable for the investigation.

T-cell epitope identification is the major step at the time of vaccine design [58]. T lymphocytes and B lymphocytes are the principal cellular components responsible for mediating an individual's immunological response [59]. Cytotoxic CD8^+^ T lymphocytes (CTL) are significant contributors to the regulation of pathogens through their ability to identify and eliminate infected cells or secrete antiviral cytokines [60]. The IEDB server was utilized to predict a substantial number of MHC-I and MHC-II peptides, specifically CTL and HTL epitopes. It has been observed that HLA-II can be utilized to make reasonable assumptions about other animals [61]. The top epitopes of MHC-I were selected depending on their ratings since a higher score denotes a more appropriate binder [15]. Furthermore, prioritize the use of adjusted rank for MHC-II. The lower adjusted score indicates the presence of suitable binders [62]. The epitopes were screened by analyzing the antigenicity score, transmembrane topology, conservancy level, toxicity profile, and allergenicity pattern. All epitopes have a high VaxiJen score and a higher level of conservancy (Table 2). The conservancy pattern guarantees that any epitope candidate vaccination will be effective over a wide range [63]. Our selected MHC-II epitopes are quite capable of inducing cytokines IFN-*γ* and IL-4 secretion is of great significance, as it suggests a harmonious Th1/Th2 immune response (Table 3). The vaccine stimulates effective antibodies, often produced by B-cells. In addition, it performs effector functions by specifically targeting foreign particles [64]. The selection of B-cell epitopes took into account factors such as antigenicity score, allergenicity, and epitope length within the range of 10–40. The B-cells that were chosen for vaccine design exhibited a higher antigenicity score (Table 4).

The selection of adjuvants and linkers plays a crucial role in amplifying the immune response and ensuring the durability of the vaccine formulations. In this investigation, adjuvants like CpG motifs were specifically opted for due to their capacity to trigger Toll-like receptor 9 (TLR-9), consequently eliciting a strong Th1 immune response, which is indispensable in the defense against intracellular pathogens such as *M. bovis*. The production of multi-epitope vaccines involved connecting the top finalized epitopes from each protein, along with appropriate linkers (EAAAK, GGGS, GPGPG, KK), adjuvants (Beta defensin-3, HABA protein, and L7/L12 ribosomal protein), and the PADRE sequence [29] (Table 5). The utilization of the PADRE sequence was frequently suggested as a means to decrease HLA polymorphism in the population [50]. Furthermore, the incorporation of adjuvants has been shown to enhance the immunogenicity of vaccine design [65].

After the vaccine construction was completed, the antigenicity and allergenicity of the constructed vaccines were determined. All vaccines demonstrated safety, were devoid of allergenic properties, and showed the ability to elicit a robust immune response (Table 6). The thermal stability of a protein is associated with its aliphatic index, whereby a higher aliphatic index signifies a greater level of thermostability for the protein [66]. Since they had a lower instability index, all vaccine constructs exhibited stability *in vivo*. The vaccine candidate bTBV3 exhibited the highest aliphatic index and the lowest instability index, suggesting that the protein demonstrated greater thermostability (Table 7). The negative GRAVY values of the vaccine constructs' indicate their hydrophilic nature [67].

In secondary structure prediction, all constructs showed a substantially similar pattern (*Supplementary 1 and Supplementary 2*). Nevertheless, it was shown that in the 3D structure, all proteins exhibited a decreased estimated RMSD (Å). In the validation study, it was observed that bTBV3 showed the highest performance among the vaccines. Specifically, 88.7% of the amino acids are located within the most favorable areas of the Ramachandran plot, while just 1.9% of the amino acids are found in the disallowed regions (Figure 4 and *Supplementary 1*). Therefore, the construct bTBV3 has the greatest concentration of amino acid residues within the region that is most preferred. Consequently, we have branded it as a promising candidate for future research.

Following validation, amino acid pairs with bond energy values less than 2.20 kcal/mol were chosen for disulfide engineering in bTBV3. From a pool of 76 pairs, it was observed that nine pairs of amino acids satisfied the criteria for disulfide engineering. The relative stability of our vaccine candidate, bTBV3, can be inferred from its possession of nine possible disulfide bonds. The relative stability of our vaccine candidate, bTBV3, can be inferred from its possession of nine possible disulfide bonds (Figure 5 and *Supplementary 1*). Subsequently, the Ellipro tool available on the IEDB website was employed to do confirmational B-cell epitope prediction in order to assess the potential for antibody generation. A total of 63 residues were found to have scores exceeding 0.8, while the remaining residues exhibited scores ranging from 0.5 to 0.76 (Figure 6 and *Supplementary 1*). Consequently, our vaccine candidate, bTBV3, demonstrates an enhanced capacity to generate a higher number of antibodies.

The docking studies provided insights into the stability and interactions of the vaccine constructs with bovine immune receptors. The strong binding affinities observed in the docking studies, with binding energy values indicating favorable interactions, suggest that the vaccine constructs can effectively engage the immune system. The binding affinity of developed bTBV3 and distinct cattle immune receptors was investigated in the docking study. The binding affinities of the bTBV3 vaccine were determined by docking with CD molecules and TLR-9. The lowest global energy was prioritized and demonstrated negative binding energy values of −40.11 and −36.63 with BoLA-A11 and TLR-9 indicating high binding affinity (Table 8 and Figure 7). The findings described in this study demonstrate a robust connection between the vaccine and MHC-I molecules, which effectively induces a potentially protective immune response.

Molecular dynamics (MD) simulations aid in the design of more stable and efficient vaccines. By studying the conformational changes and interactions within the vaccine components, MD simulations contribute to understanding the structural dynamics of proteins and their complexes, essential for vaccine efficacy. According to the MD simulation study, it was shown that the complex exhibited eigenvalues of 2.318704e-06, suggesting a reduced likelihood of deformability. Therefore, based on the deformability graph, it can be observed that the complex exhibits reduced deformability for every residue. The insignificance of hinge locations in the chain was determined through analysis, which subsequently confirmed a minimal likelihood of deformability for every residue. This finding further supports our prediction, as illustrated in Figure 8. The *in silico* immune simulation study confirmed the immune cell response to antigen clearance. After the administration of the vaccine, the observed elevation of IgM production signifies the initiation of a primary immune response. Memory development is accompanied by an increase in the population of Th (helper) cells (Figure 9).

Furthermore, to ensure protein expression, the vector was selected and designed for optimal expression in *E. coli*, along with the assessment of the codon adaptation index (CAI) and GC content. The CAI value and GC content promote the efficacy, stability, and increased yields of the recombinant protein. This optimization is essential for producing sufficient quantities of the vaccine antigen. In this investigation, the codon adaptation analysis revealed a notable increase in both the relative adaptiveness and GC content (*Supplementary 2*). The nucleotide sequence of the vaccine was subsequently cloned using *in silico* restriction analysis to evaluate bTBV3's compatibility with the pET28a (+) vector, and it is deemed to be very suitable for efficient gene production. The CAI value and the GC content showed a successful cloning process with high levels of protein expression within the host bacterium (Figure 10).

## 5. Conclusion

The findings of this study present a significant advancement in the field of bovine tuberculosis (bTB) vaccine development. The novel multi-epitope vaccine candidate bTBV3 has demonstrated robust interactions with Toll-like receptors, exhibiting high stability in computational simulations. The stability analysis revealed low deformability, and immune simulations indicated a strong primary immune response characterized by elevated IgM levels and an increase in Th cell populations, suggesting effective memory formation. Codon optimization and *in silico* cloning confirmed the vaccine's potential for high protein expression in host bacteria. These promising results warrant further *in vitro* and *in vivo* experimentation to validate bTBV3's efficacy as a subunit vaccine against bTB.

## Figures and Tables

**Table 1:** List of amino acid sequences of selected MPB83, ArfA, DnaK, GrpE, and LpqH proteins.

Sl. no.	Name of proteins	Accession number	Amino acid sequence
1	Cell surface glycolipoprotein MPB83	P0CAX7	MINVQAKPAAAASLAAIAIAFLAGCSSTKPVSQDTSPKPATSPAAPVTTAAMADPAADLI GRGCAQYAAQNPTGPGSVAGMAQDPVATAASNNPMLSTLTSALSGKLNPDVNLVDTLNGG EYTVFAPTNAAFDKLPAATIDQLKTDAKLLSSILTYHVIAGQASPSRIDGTHQTLQGADL TVIGARDDLMVNNAGLVCGGVHTANATVYMIDTVLMPPAQ

2	Peptidoglycan-binding protein ArfA	A1KH31	MASKAGLGQTPATTDARRTQKFYRGSPGRPWLIGAVVIPLLIAAIGYGAFERPQSVTGPT GVLPTLTPTSTRGASALSLSLLSISRSGNTVTLIGDFPDEAAKAALMTALNGLLAPGVNV IDQIHVDPVVRSLDFSSAEPVFTASVPIPDFGLKVERDTVTLTGTAPSSEHKDAVKRAAT STWPDMKIVNNIEVTGQAPPGPPASGPCADLQSAINAVTGGPIAFGNDGASLIPADYEIL NRVADKLKACPDARVTINGYTDNTGSEGINIPLSAQRAKIVADYLVARGVAGDHIATVGL GSVNPIASNATPEGRAKNRRVEIVVN

3	Chaperone protein DnaK	P0A5C0	MARAVGIDLGTTNSVVSVLEGGDPVVVANSEGSRTTPSIVAFARNGEVLVGQPAKNQAVT NVDRTVRSVKRHMGSDWSIEIDGKKYTAPEISARILMKLKRDAEAYLGEDITDAVITTPA YFNDAQRQATKDAGQIAGLNVLRIVNEPTAAALAYGLDKGEKEQRILVFDLGGGTFDVSL LEIGEGVVEVRATSGDNHLGGDDWDQRVVDWLVDKFKGTSGIDLTKDKMAMQRLREAAEK AKIELSSSQSTSINLPYITVDADKNPLFLDEQLTRAEFQRITQDLLDRTRKPFQSVIADT GISVSEIDHVVLVGGSTRMPAVTDLVKELTGGKEPNKGVNPDEVVAVGAALQAGVLKGEV KDVLLLDVTPLSLGIETKGGVMTRLIERNTTIPTKRSETFTTADDNQPSVQIQVYQGERE IAAHNKLLGSFELTGIPPAPRGIPQIEVTFDIDANGIVHVTAKDKGTGKENTIRIQEGSG LSKEDIDRMIKDAEAHAEEDRKRREEADVRNQAETLVYQTEKFVKEQREAEGGSKVPEDT LNKVDAAVAEAKAALGGSDISAIKSAMEKLGQESQALGQAIYEAAQAASQATGAAHPGGE PGGAHPGSADDVVDAEVVDDGREAK

4	Protein GrpE	A1KFH3	MTDGNQKPDGNSGEQVTVTDKRRIDPETGEVRHVPPGDMPGGTAAADAAHTEDKVAELTA DLQRVQADFANYRKRALRDQQAAADRAKASVVSQLLGVLDDLERARKHGDLESGPLKSVA DKLDSALTGLGLVAFGAEGEDFDPVLHEAVQHEGDGGQGSKPVIGTVMRQGYQLGEQVLR HALVGVVDTVVVDAAELESVDDGTAVADTAENDQADQGNSADTLGEQAESEPSGS

5	Lipoprotein LpqH	A0A0H3M9Z0	MKRGLTVAVAGAAILVAGLSGCSSNKSTTGSGETTTAAGTTASPGAASGPKVVIDGKDQN VTGSVVCTTAAGNVNIAIGGAATGIAAVLTDGNPPEVKSVGLGNVNGVTLGYTSGTGQGN ASATKDGSHYKITGTATGVDMANPMSPVNKSFEIEVTCS

**Table 2:** Lists of selected T-cell epitopes, antigenicity, allergenicity, topology, toxicity, and conservancy score.

Type	Protein	Epitopes	Antigenicity	Allergenicity	Topology	Toxicity	Conservancy (%)	Remarks
MHC-I	Cell surface glycolipoprotein MPB83	TGPGSVAGM	1.28	Nonallergen	Outside	Nontoxin	85.71	Selected
		LAAIAIAFL	1.1352	Nonallergen	Outside	Nontoxin	42.86	Not selected
		SQDTSPKPA	1.0405	Nonallergen	Outside	Nontoxin	85.71	Selected
		SLAAIAIAF	1.039	Nonallergen	Outside	Nontoxin	42.86	Not selected
		GKLNPDVNL	0.9777	Nonallergen	Outside	Nontoxin	85.71	Not selected
	Peptidoglycan-binding protein ArfA	VVIPLLIAA	1.5286	Nonallergen	Outside	Nontoxin	57.14	Not selected
		VIPLLIAAI	1.3697	Nonallergen	Outside	Nontoxin	71.43	Selected
		GASALSLSL	1.2465	Nonallergen	Outside	Nontoxin	71.43	Selected
		SVPIPDFGL	1.1761	Nonallergen	Outside	Nontoxin	57.14	Not selected
		GAVVIPLLI	1.1721	Nonallergen	Outside	Nontoxin	57.14	Not selected
	Chaperone protein DnaK	GGGTFDVSL	1.7383	Nonallergen	Outside	Nontoxin	83.33	Selected
		STSINLPYI	1.2953	Nonallergen	Outside	Nontoxin	83.33	Selected
		LLDVTPLSL	1.0021	Nonallergen	Outside	Nontoxin	83.33	Not selected
		QSTSINLPY	0.9888	Nonallergen	Outside	Nontoxin	83.33	Not selected
		GSKVPEDTL	0.8004	Nonallergen	Outside	Nontoxin	66.67	Not selected
	Protein GrpE	GNSGEQVTV	1.9971	Nonallergen	Outside	Nontoxin	42.86	Not selected
		GGQGSKPVI	1.9407	Nonallergen	Outside	Nontoxin	85.71	Selected
		ESVDDGTAV	0.9464	Nonallergen	Outside	Nontoxin	85.71	Selected
		SVDDGTAVA	0.8491	Nonallergen	Outside	Nontoxin	85.71	Not selected
		DSALTGLGL	0.716	Nonallergen	Outside	Nontoxin	85.71	Not selected
	Lipoprotein LpqH	STTGSGETT	3.1686	Nonallergen	Outside	Nontoxin	60	Selected
		GTGQGNASA	2.1536	Nonallergen	Outside	Nontoxin	60	Selected
		LTDGNPPEV	1.191	Nonallergen	Outside	Nontoxin	60	Not selected
		AIGGAATGI	1.1858	Nonallergen	Outside	Nontoxin	60	Not selected
		LGNVNGVTL	1.0494	Nonallergen	Outside	Nontoxin	60	Not selected
MHC-II	Cell surface glycolipoprotein MPB83	IAIAFLAGCSST	1.0748	Nonallergen	Outside	Nontoxin	42.86	Not selected
		KLNPDVNLVDTL	0.9319	Nonallergen	Outside	Nontoxin	85.71	Selected
		DVNLVDTLNGGE	0.8499	Nonallergen	Outside	Nontoxin	85.71	Selected
		VSQDTSPKPATS	0.8073	Nonallergen	Outside	Nontoxin	85.71	Not selected
		AIAIAFLAGCSS	0.7885	Nonallergen	Outside	Nontoxin	42.86	Not selected
	Peptidoglycan-binding protein ArfA	VAGDHIATVGLG	1.1015	Nonallergen	Outside	Nontoxin	71.43	Selected
		GVAGDHIATVGL	1.0828	Nonallergen	Outside	Nontoxin	71.43	Selected
		AVVIPLLIAAIG	0.9839	Nonallergen	Outside	Nontoxin	57.14	Nonselected
		WLIGAVVIPLLI	0.9588	Nonallergen	Outside	Nontoxin	57.14	Nonselected
		VVIPLLIAAIGY	0.9571	Nonallergen	Outside	Nontoxin	57.14	Nonselected
	Chaperone protein DnaK	PVVVANSEGSRT	1.5284	Nonallergen	Outside	Nontoxin	83.33	Selected
		DPVVVANSEGSR	1.3484	Nonallergen	Outside	Nontoxin	83.33	Selected
		GIDLGTTNSVVS	1.1834	Nonallergen	Outside	Nontoxin	66.67	Not selected
		ILVFDLGGGTFD	1.0637	Nonallergen	Outside	Nontoxin	83.33	Not selected
		DVLLLDVTPLSL	0.9816	Nonallergen	Outside	Nontoxin	83.33	Not selected
	Protein GrpE	VLHEAVQHEGDG	0.9832	Nonallergen	Outside	Nontoxin	100	Selected
		VDTVVVDAAELE	0.9045	Nonallergen	Outside	Nontoxin	85.71	Selected
		DTVVVDAAELES	0.8153	Nonallergen	Outside	Nontoxin	85.71	Not selected
		VVVDAAELESVD	0.79	Nonallergen	Outside	Nontoxin	85.71	Not selected
		VGVVDTVVVDAA	0.66	Nonallergen	Outside	Nontoxin	85.71	Not selected
	Lipoprotein LpqH	TLGYTSGTGQGN	1.9176	Nonallergen	Outside	Nontoxin	60	Selected
		LGYTSGTGQGNA	1.8231	Nonallergen	Outside	Nontoxin	60	Selected
		VTLGYTSGTGQG	1.797	Nonallergen	Outside	Nontoxin	60	Not selected
		GLSGCSSNKSTT	1.5889	Nonallergen	Outside	Nontoxin	60	Not selected
		GVTLGYTSGTGQ	1.5021	Nonallergen	Outside	Nontoxin	60	Not selected

**Table 3:** Cytokines induction capabilities of selected MHC-II epitopes.

MHC-II epitopes	IFN- *γ*	IL-4
KLNPDVNLVDTL	Noninducer	Noninducer
DVNLVDTLNGGE	Noninducer	Noninducer
VAGDHIATVGLG	Inducer	Inducer
GVAGDHIATVGL	Noninducer	Inducer
PVVVANSEGSRT	Noninducer	Noninducer
DPVVVANSEGSR	Noninducer	Inducer
VLHEAVQHEGDG	Noninducer	Inducer
VDTVVVDAAELE	Inducer	Inducer
TLGYTSGTGQGN	Noninducer	Inducer
LGYTSGTGQGNA	Noninducer	Inducer

**Table 4:** B-cell epitopes by bepipred linear epitope prediction method.

Protein	Epitope	Antigenicity	Allergenicity
Cell surface glycolipoprotein MPB83	QASPSRIDGTHQTLQ	1.0949	Nonallergen
Peptidoglycan-binding protein ArfA	AGLGQTPATTDARRTQKFYRGSPG	0.9293	Nonallergen
Chaperone protein DnaK	RHMGSDWSIEIDGKK	1.5424	Nonallergen
Protein GrpE	NQKPDGNSGEQVTVTDKRRIDPETGEVRHVPPGDMPGG	1.2546	Allergen
Lipoprotein LpqH	SSNKSTTGSGETTTAAGTTASPGAASG	2.0146	Nonallergen

**Table 5:** Vaccine constructs bTBV1, bTBV2, and bTBV3 and their CTL, HTL, and HCL with defensing adjuvant and PADRE sequence.

Vaccine construct	Compositions	Candidate vaccine protein sequence	No. of amino acids
bTBV1	Predicted CTL, HTL, and BCL epitopes with defensing adjuvant and PADRE sequence	**EAAAK**GIINTLQKYYCRVRGGRCAVLSCLPKEEQIGKCSTRGRKCCRRKK**EAAAK**AKFVAAWTLKAAA**GGGS**TGPGSVAGM**GGGS**SQDTSPKPA**GGGS**VIPLLIAAI**GGGS**GASALSLSL**GGGS**GGGTFDVSL**GGGS**STSINLPYI**GGGS**GGQGSKPVI**GGGS**ESVDDGTAV**GGGS**STTGSGETT**GGGS**GTGQGNASA**GPGPG**KLNPDVNLVDTL**GPGPG**DVNLVDTLNGGE**GPGPG**VAGDHIATVGLG**GPGPG**GVAGDHIATVGL**GPGPG**PVVVANSEGSRT**GPGPG**DPVVVANSEGSR**GPGPG**VLHEAVQHEGDG**GPGPG**VDTVVVDAAELE**GPGPG**TLGYTSGTGQGN**GPGPG**LGYTSGTGQGNA**KK**QASPSRIDGTHQTLQ**KK**AGLGQTPATTDARRTQKFYRGSPG**KK**RHMGSDWSIEIDGKK**KK**NQKPDGNSGEQVTVTDKRRIDPETGEVRHVPPGDMPGG**KK**SSNKSTTGSGETTTAAGTTASPGAASG**KK**AKFVAAWTLKAAA**GGGS**	516

bTBV2	Predicted CTL, HTL, and BCL epitopes with HABA adjuvant and PADRE sequence	**EAAAK**MAENPNIDDLPAPLLAALGAADLALATVNDLIANLRERAEETRAETRTRVEERRARLTKFQEDLPEQFIELRDKFTTEELRKAAEGYLEAATNRYNELVERGEAALQRLRSQTAFEDASARAEGYVDQAVELTQEALGTVASQTRAVGERAAKLVGIELPGKAEAAGKKAQKAIAKAPAKKASAKKAPAKKAPAKKAAAKKVTQK**EAAAK**AKFVAAWTLKAAA**GGGS**TGPGSVAGM**GGGS**SQDTSPKPA**GGGS**VIPLLIAAI**GGGS**GASALSLSL**GGGS**GGGTFDVSL**GGGS**STSINLPYI**GGGS**GGQGSKPVI**GGGS**ESVDDGTAV**GGGS**STTGSGETT**GGGS**GTGQGNASA**GPGPG**KLNPDVNLVDTL**GPGPG**DVNLVDTLNGGE**GPGPG**VAGDHIATVGLG**GPGPG**GVAGDHIATVGL**GPGPG**PVVVANSEGSRT**GPGPG**DPVVVANSEGSR**GPGPG**VLHEAVQHEGDG**GPGPG**VDTVVVDAAELE**GPGPG**TLGYTSGTGQGN**GPGPG**LGYTSGTGQGNA**KK**QASPSRIDGTHQTLQ**KK**AGLGQTPATTDARRTQKFYRGSPG**KK**RHMGSDWSIEIDGKK**KK**NQKPDGNSGEQVTVTDKRRIDPETGEVRHVPPGDMPGG**KK**SSNKSTTGSGETTTAAGTTASPGAASG**KK**AKFVAAWTLKAAA**GGGS**	676

bTBV3	Predicted CTL, HTL, and BCL epitopes with L7/L12 ribosomal protein adjuvant and PADRE sequence	**EAAAK**MAKLSTDELLDAFKEMTLLELSDFVKKFEETFEVTAAAPVAVAAAGAAPAGAAVEAAEEQSEFDVILEAAGDKKIGVIKVVREIVSGLGLKEAKDLVDGAPKPLLEKVAKEAADEAKAKLEAAGATVTVK**EAAAK**AKFVAAWTLKAAA**GGGS**TGPGSVAGM**GGGS**SQDTSPKPA**GGGS**VIPLLIAAI**GGGS**GASALSLSL**GGGS**GGGTFDVSL**GGGS**STSINLPYI**GGGS**GGQGSKPVI**GGGS**ESVDDGTAV**GGGS**STTGSGETT**GGGS**GTGQGNASA**GPGPG**KLNPDVNLVDTL**GPGPG**DVNLV>DTLNGGE**GPGPG**VAGDHIATVGLG**GPGPG**GVAGDHIATVGL**GPGPG**PVVVANSEGSRT**GPGPG**DPVVVANSEGSR**GPGPG**VLHEAVQHEGDG**GPGPG**VDTVVVDAAELE**GPGPG**TLGYTSGTGQGN**GPGPG**LGYTSGTGQGNA**KK**QASPSRIDGTHQTLQ**KK**AGLGQTPATTDARRTQKFYRGSPG**KK**RHMGSDWSIEIDGKK**KK**NQKPDGNSGEQVTVTDKRRIDPETGEVRHVPPGDMPGG**KK**SSNKSTTGSGETTTAAGTTASPGAASG**KK**AKFVAAWTLKAAA**GGGS**	601

Bold highlights the linkers.

**Table 6:** List of antigenicity, allergenicity, and solubility of the vaccine constructs.

Vaccine construct	Antigenicity	Allergenicity	Solubility
bTBV1	1.5364	Nonallergen	0.604
bTBV2	1.3309	Nonallergen	0.565
bTBV3	1.3521	Nonallergen	0.567

**Table 7:** Physiochemical properties of the vaccine bTBV1, bTBV2, and bTBV3.

Vaccine candidate	Molecular weight	Theoretical pI	Extinction coefficients	Estimated half-life (*Escherichia coli*, *in vivo*) (hr)	Instability index	Aliphatic index	GRAVY
bTBV1	49,426.49	9.20	25,815	>10	30.57	58.45	−0.453
bTBV2	66,441.49	8.78	26,930	>10	32.75	65.09	−0.478
bTBV3	57,705.78	5.41	22,460	>10	27.26	67.60	−0.298

**Table 8:** List of molecular docking scores of the vaccine constructs bTBV3.

PDB ID	Score	Area	Global energy	ACE	HB
3PWV	17,146	2,570.00	−40.11	0.79	−2.31
5EBG	17,786	3,468.70	−3.89	−0.58	−0.42
5Y3M	17,538	2,343.50	−36.63	0.81	−5.08

## Data Availability

All data generated and analyzed in this study are included in the manuscript and supplementary files.

## Supplementary Materials

Supplementary 1 Table 1: the antigenicity score, allergenicity, and topology of the selected five proteins. Table 2: physiochemical properties of selected five proteins. Table 3: MHC-I and MHC-II epitopes of Cell surface glycolipoprotein MPB83. Table 4: MHC-I and MHC-II epitopes of Peptidoglycan-binding protein ArfA. Table 5: MHC-I and MHC-II epitopes of Chaperone protein DnaK. Table 6: MHC-I and MHC-II epitopes of Protein GrpE. Table 7: MHC-I and MHC-II epitopes of Lipoprotein LpqH. Table 8: secondary structure analysis of the vaccine candidates. Table 9: validation score (Ramachandran plot) for bTBV1, bTBV2, and bTBV3 construct. Table 10: protein disulfide engineering score of bTBV3 vaccine construct. Table 11: a list of the discontinuous B-cell epitopes predicted by the ElliPro server.

Supplementary 2 Figure 1: secondary structure predictions of vaccine constructs. (a) bTBV1, (b) bTBV2, and (c) bTBV3. Figure 2: validation map of (a) bTBV1 and (b) bTBV2 in a Ramachandran plot. Figure 3: adaptiveness of vaccine constructs bTBV3 in *E. coli* strain K12.

## References

[1] Mareledwane V. E., Adesiyun A. A., Thompson P. N., Hlokwe T. M. (2022). Application of the gamma-interferon assay to determine the prevalence of bovine tuberculosis in slaughter livestock at abattoirs in Gauteng, South Africa. *Veterinary Medicine and Science*.

[2] Islam M. N., Khan M. K., Khan M. F. R., Kostoulas P., Rahman A. K. M. A., Alam M. M. (2021). Risk factors and true prevalence of bovine tuberculosis in Bangladesh. *PLoS ONE*.

[3] Hossain M. B., Sayeed A., Faruk S. A., Khan M., Rumi A., Hoque A. (2023). Sero-epidemiology of bovine tuberculosis in dairy cattle in Chattogram, Bangladesh. *Turkish Journal of Veterinary Research*.

[4] Agbalaya M. A., Ishola O. O., Adesokan H. K., Fawole O. I. (2020). Prevalence of bovine tuberculosis in slaughtered cattle and factors associated with risk of disease transmission among cattle handlers at Oko-Oba Abattoir, Lagos, Nigeria. *Veterinary World*.

[5] Kumari R S., Sethi G., Krishna R. (2023). Development of multi-epitope based subunit vaccine against *Mycobacterium Tuberculosis* using immunoinformatics approach. *Journal of Biomolecular Structure and Dynamics*.

[6] Dhakshinamoorthy D. R., Karuppasamy R. (2023). Novel multi-epitope vaccine design against *Mycobacterium tuberculosis*: an Immunoinformatics strategy. *Research Journal of Biotechnology*.

[7] Ning Y., Cai Y., Liu X., Gu C., Meng X., Qiao J. (2023). A multi-stage and multi-epitope vaccine against Mycobacterium tuberculosis based on an immunoinformatics approach. *Chinese Journal of Cellular and Molecular Immunology*.

[8] Uddin M. B., Tanni F. Y., Hoque S. F. (2022). A candidate multi-epitope vaccine against Lumpy skin disease. *Transboundary and Emerging Diseases*.

[9] Moliva J. I., Turner J., Torrelles J. B. (2015). Prospects in *Mycobacterium bovis* bacille calmette et guérin (BCG) vaccine diversity and delivery: why does BCG fail to protect against tuberculosis?. *Vaccine*.

[10] Brandt L., Feino Cunha J., Weinreich Olsen A. (2002). Failure of the *Mycobacterium bovis* BCG vaccine: some species of environmental mycobacteria block multiplication of BCG and induction of protective immunity to tuberculosis. *Infection and Immunity*.

[11] Brosch R., Gordon S. V., Garnier T. (2007). Genome plasticity of BCG and impact on vaccine efficacy. *Proceedings of the National Academy of Sciences*.

[12] Mangtani P., Abubakar I., Ariti C. (2014). Protection by BCG vaccine against tuberculosis: a systematic review of randomized controlled trials. *Clinical Infectious Diseases*.

[13] Sayers E. W., Beck J., Bolton E. E. (2021). Database resources of the national center for biotechnology information. *Nucleic Acids Research*.

[14] The UniProt Consortium (2021). UniProt: the universal protein knowledgebase in 2021. *Nucleic Acids Research*.

[15] Reynisson B., Alvarez B., Paul S., Peters B., Nielsen M. (2020). NetMHCpan-4.1 and NetMHCIIpan-4.0: improved predictions of MHC antigen presentation by concurrent motif deconvolution and integration of MS MHC eluted ligand data. *Nucleic Acids Research*.

[16] Farrell D., Jones G., Pirson C. (2016). Integrated computational prediction and experimental validation identifies promiscuous T cell epitopes in the proteome of *Mycobacterium bovis*. *Microbial Genomics*.

[17] Sturniolo T., Bono E., Ding J. (1999). Generation of tissue-specific and promiscuous HLA ligand databases using DNA microarrays and virtual HLA class II matrices. *Nature Biotechnology*.

[18] Doytchinova I. A., Flower D. R. (2007). VaxiJen: a server for prediction of protective antigens, tumour antigens and subunit vaccines. *BMC Bioinformatics*.

[19] Dimitrov I., Bangov I., Flower D. R., Doytchinova I. (2014). AllerTOP v.2—a server for in silico prediction of allergens. *Journal of Molecular Modeling*.

[20] Dimitrov I., Naneva L., Doytchinova I., Bangov I. (2014). AllergenFP: allergenicity prediction by descriptor fingerprints. *Bioinformatics*.

[21] Bui H.-H., Sidney J., Li W., Fusseder N., Sette A. (2007). Development of an epitope conservancy analysis tool to facilitate the design of epitope-based diagnostics and vaccines. *BMC Bioinformatics*.

[22] Gish W., States D. J. (1993). Identification of protein coding regions by database similarity search. *Nature Genetics*.

[23] Madden T. L., Tatusov R. L., Zhang J. (1996). Applications of network BLAST server. *Methods in Enzymology*.

[24] Sharma N., Naorem L. D., Jain S., Raghava G. P. S. (2022). ToxinPred2: an improved method for predicting toxicity of proteins. *Briefings in Bioinformatics*.

[25] Thomsen M., Lundegaard C., Buus S., Lund O., Nielsen M. (2013). MHCcluster, a method for functional clustering of MHC molecules. *Immunogenetics*.

[26] Dhanda S. K., Vir P., Raghava G. P. S. (2013). Designing of interferon-gamma inducing MHC class-II binders. *Biology Direct*.

[27] Dhanda S. K., Gupta S., Vir P., Raghava G. P. S. (2013). Prediction of IL4 inducing peptides. *Clinical and Developmental Immunology*.

[28] Jespersen M. C., Peters B., Nielsen M., Marcatili P. (2017). BepiPred-2.0: improving sequence-based B-cell epitope prediction using conformational epitopes. *Nucleic Acids Research*.

[29] Mahmoodi S., Amirzakaria J. Z., Ghasemian A. (2023). *In silico* design and validation of a novel multi-epitope vaccine candidate against structural proteins of Chikungunya virus using comprehensive immunoinformatics analyses. *PLoS ONE*.

[30] Rana A., Akhter Y. (2016). A multi-subunit based, thermodynamically stable model vaccine using combined immunoinformatics and protein structure based approach. *Immunobiology*.

[31] Krogh A., Larsson B., von Heijne G., Sonnhammer E. L. L. (2001). Predicting transmembrane protein topology with a hidden markov model: application to complete genomes. *Journal of Molecular Biology*.

[32] Tusnády G. E., Simon I. (1998). Principles governing amino acid composition of integral membrane proteins: application to topology prediction. *Journal of Molecular Biology*.

[33] Hebditch M., Carballo-Amador M. A., Charonis S., Curtis R., Warwicker J. (2017). Protein–Sol: a web tool for predicting protein solubility from sequence. *Bioinformatics*.

[34] Duvaud S., Gabella C., Lisacek F., Stockinger H., Ioannidis V., Durinx C. (2021). Expasy, the swiss bioinformatics resource portal, as designed by its users. *Nucleic Acids Research*.

[35] Buchan D. W. A., Jones D. T. (2019). The PSIPRED protein analysis workbench: 20 years on. *Nucleic Acids Research*.

[36] Xu J., McPartlon M., Li J. (2021). Improved protein structure prediction by deep learning irrespective of co-evolution information. *Nature Machine Intelligence*.

[37] Heo L., Park H., Seok C. (2013). GalaxyRefine: protein structure refinement driven by side-chain repacking. *Nucleic Acids Research*.

[38] Laskowski R. A., MacArthur M. W., Moss D. S., Thornton J. M. (1993). PROCHECK: a program to check the stereochemical quality of protein structures. *Journal of Applied Crystallography*.

[39] Craig D. B., Dombkowski A. A. (2013). Disulfide by design 2.0: a web-based tool for disulfide engineering in proteins. *BMC Bioinformatics*.

[40] Ponomarenko J., Bui H.-H., Li W. (2008). ElliPro: a new structure-based tool for the prediction of antibody epitopes. *BMC Bioinformatics*.

[41] (2012). Jmol: an open-source Java viewer for chemical structures in 3D. http://www.jmol.org/.

[42] Burley S. K., Bhikadiya C., Bi C. (2021). RCSB Protein Data Bank: powerful new tools for exploring 3D structures of biological macromolecules for basic and applied research and education in fundamental biology, biomedicine, biotechnology, bioengineering and energy sciences. *Nucleic Acids Research*.

[43] Mashiach E., Schneidman-Duhovny D., Peri A., Shavit Y., Nussinov R., Wolfson H. J. (2010). An integrated suite of fast docking algorithms. *Proteins: Structure, Function, and Bioinformatics*.

[44] Mashiach E., Schneidman-Duhovny D., Andrusier N., Nussinov R., Wolfson H. J. (2008). FireDock: a web server for fast interaction refinement in molecular docking. *Nucleic Acids Research*.

[45] Yan Y., Tao H., He J., Huang S.-Y. (2020). The HDOCK server for integrated protein–protein docking. *Nature Protocols*.

[46] López-Blanco J. R., Aliaga J. I., Quintana-Ortí E. S., Chacón P. (2014). iMODS: internal coordinates normal mode analysis server. *Nucleic Acids Research*.

[47] Rapin N., Lund O., Bernaschi M., Castiglione F. (2010). Computational immunology meets bioinformatics: the use of prediction tools for molecular binding in the simulation of the immune system. *PLoS ONE*.

[48] Abraham Peele K., Srihansa T., Krupanidhi S., Ayyagari V. S., Venkateswarulu T. C. (2021). Design of multi-epitope vaccine candidate against SARS-CoV-2: a *in-silico* study. *Journal of Biomolecular Structure and Dynamics*.

[49] Grote A., Hiller K., Scheer M. (2005). JCat: a novel tool to adapt codon usage of a target gene to its potential expression host. *Nucleic Acids Research*.

[50] Ghaffari-Nazari H., Tavakkol-Afshari J., Jaafari M. R. (2015). Improving multi-epitope long peptide vaccine potency by using a strategy that enhances CD4+ T Help in BALB/c mice. *PLoS ONE*.

[51] Solanki V., Tiwari V. (2018). Subtractive proteomics to identify novel drug targets and reverse vaccinology for the development of chimeric vaccine against *Acinetobacter baumannii*. *Scientific Reports*.

[52] Wu F., Xu J. (2021). Deep template-based protein structure prediction. *PLoS Computational Biology*.

[53] Chambers M. A., Whelan A. O., Spallek R. (2010). Non-acylated *Mycobacterium bovis* glycoprotein MPB83 binds to TLR1/2 and stimulates production of matrix metalloproteinase 9. *Biochemical and Biophysical Research Communications*.

[54] Yao Y., Barghava N., Kim J., Niederweis M., Marassi F. M. (2012). Molecular structure and peptidoglycan recognition of mycobacterium tuberculosis ArfA (Rv0899). *Journal of Molecular Biology*.

[55] Chuang Y.-M., Pinn M. L., Karakousis P. C., Hung C.-F. (2018). Intranasal immunization with DnaK protein induces protective mucosal immunity against tuberculosis in CD4-depleted mice. *Frontiers in Cellular and Infection Microbiology*.

[56] Kim W. S., Jung I. D., Kim J.-S. (2018). *Mycobacterium tuberculosis* GrpE, A heat-shock stress responsive chaperone, promotes Th1-biased T cell immune response via TLR4-mediated activation of dendritic cells. *Frontiers in Cellular and Infection Microbiology*.

[57] Sánchez A., Espinosa P., García T., Mancilla R. (2012). The 19 kDa *Mycobacterium tuberculosis* lipoprotein (LpqH) induces macrophage apoptosis through extrinsic and intrinsic pathways: a role for the mitochondrial apoptosis-inducing factor. *Clinical and Developmental Immunology*.

[58] Amorim K. N. S., Rampazo E. V., Antonialli R. (2016). The presence of T cell epitopes is important for induction of antibody responses against antigens directed to DEC205^+^ dendritic cells. *Scientific Reports*.

[59] Sarkar B., Ullah M. A., Araf Y. (2020). A systematic and reverse vaccinology approach to design novel subunit vaccines against Dengue virus type-1 (DENV-1) and human Papillomavirus-16 (HPV-16). *Informatics in Medicine Unlocked*.

[60] Garcia K. C., Teyton L., Wilson I. A. (1999). Structural basis of T cell recognition. *Annual Review of Immunology*.

[61] Ros-Lucas A., Correa-Fiz F., Bosch-Camós L., Rodriguez F., Alonso-Padilla J. (2020). Computational analysis of african swine fever virus protein space for the design of an epitope-based vaccine ensemble. *Pathogens*.

[62] Wang P., Sidney J., Dow C., Mothé B., Sette A., Peters B. (2008). A systematic assessment of MHC class II peptide binding predictions and evaluation of a consensus approach. *PLoS Computational Biology*.

[63] Hasan M., Ghosh P. P., Azim K. F. (2019). Reverse vaccinology approach to design a novel multi-epitope subunit vaccine against avian influenza A (H7N9) virus. *Microbial Pathogenesis*.

[64] Cooper N. R., Nemerow G. R. (1984). The role of antibody and complement in the control of viral infections. *Journal of Investigative Dermatology*.

[65] Yang Y., Sun W., Guo J. (2015). *In silico* design of a DNA-based HIV-1 multi-epitope vaccine for Chinese populations. *Human Vaccines & Immunotherapeutics*.

[66] Panda S., Chandra G. (2012). Physicochemical characterization and functional analysis of some snake venom toxin proteins and related non-toxin proteins of other chordates. *Bioinformation*.

[67] Chang K. Y., Yang J.-R., Isalan M. (2013). Analysis and prediction of highly effective antiviral peptides based on random forests. *PLoS ONE*.

