# Supplementary material for: In Silico Driven Multi-Epitope Subunit Candidate Vaccine against Bovine Tuberculosis
Source: Transbound Emerg Dis. 2024 Sep 4;2024:5534041. doi: 10.1155/2024/5534041 (PMC12016833; doi:10.1155/2024/5534041)
Supplement: Supplementary 1 — Table 1: the antigenicity score, allergenicity, and topology of the selected five proteins. Table 2: physiochemical properties of selected five proteins. Table 3: MHC-I and MHC-II epitopes of Cell surface glycolipoprotein MPB83. Table 4: MHC-I and MHC-II epitopes of Peptidoglycan-binding protein ArfA. Table 5: MHC-I and MHC-II epitopes of Chaperone protein DnaK. Table 6: MHC-I and MHC-II epitopes of Protein GrpE. Table 7: MHC-I and MHC-II epitopes of Lipoprotein LpqH. Table 8: secondary structure analysis of the vaccine candidates. Table 9: validation score (Ramachandran plot) for bTBV1, bTBV2, and bTBV3 construct. Table 10: protein disulfide engineering score of bTBV3 vaccine construct. Table 11: a list of the discontinuous B-cell epitopes predicted by the ElliPro server. [file 5534041.f1.docx]

**Table S1.** The antigenicity score, allergenicity, and topology of the selected five proteins.

| **Proteins** | **Uniprot accession number** | **Antigenicity** | **Allergenicity** | **Topology** |
| --- | --- | --- | --- | --- |
| Cell surface glycolipoprotein MPB83 | P0CAX7 | 0.5742 | Non-allergen | Outside |
| Peptidoglycan-binding protein ArfA | A1KH31 | 0.6056 | Non-allergen | Outside |
| Chaperone protein DnaK | P0A5C0 | 0.6417 | Non-allergen | Outside |
| Protein GrpE | A1KFH3 | 0.7949 | Non-allergen | Outside |
| Lipoprotein LpqH | A0A0H3M9Z0 | 1.1519 | Non-allergen | Outside |

**Table S2.** Physiochemical properties of selected five proteins.

| **Protein** | **No. of Amino Acids** | **Molecular wt** | **Instability Index** | **Aliphatic Index** | **Gravy value** | **Half life**  **(In *E.coli*)** | **Theoretical pI** | **Extinction co-efficient** |
| --- | --- | --- | --- | --- | --- | --- | --- | --- |
| Cell surface glycolipoprotein MPB83 | 220 | 22070.06 | 30.50 | 89.82 | 0.188 | >10 hours | 4.86 | 6085 |
| Peptidoglycan-binding protein ArfA | 326 | 33574.15 | 31.15 | 94.02 | 0.035 | >10 hours | 6.84 | 18575 |
| Chaperone protein DnaK | 625 | 66830.86 | 28.37 | 88.34 | -0.368 | >10 hours | 4.85 | 28420 |
| Protein GrpE | 235 | 24558.69 | 20.04 | 77.23 | -0.561 | >10 hours | 4.39 | 2980 |
| Lipoprotein LpqH | 159 | 15146.87 | 24.50 | 76.10 | 0.112 | >10 hours | 6.53 | 3105 |

**Table S3.** MHC-I and MHC-II epitopes of Cell surface glycolipoprotein MPB83.

| **Type** | **Allele** | **Start** | **End** | **Peptide** | **Antigenicity** | **Topology** |
| --- | --- | --- | --- | --- | --- | --- |
| MHC-I | BoLA-3:00201 | 13 | 21 | TGPGSVAGM | 1.28 | Outside |
|  | BoLA-3:00201 | 14 | 22 | LAAIAIAFL | 1.1352 | Outside |
|  | BoLA-2:00601 | 32 | 40 | SQDTSPKPA | 1.0405 | Outside |
|  | BoLA-5:00301 | 13 | 21 | SLAAIAIAF | 1.039 | Outside |
|  | BoLA-5:00301 | 45 | 53 | GKLNPDVNL | 0.9777 | Outside |
|  | BoLA-3:00401 | 34 | 42 | DTSPKPATS | 0.9486 | Outside |
|  | BoLA-2:00501 | 36 | 44 | SPKPATSPA | 0.7229 | Outside |
|  | BoLA-3:00401 | 35 | 43 | TSPKPATSP | 0.711 | Outside |
|  | BoLA-2:00501 | 15 | 23 | LPAATIDQL | 0.6835 | Outside |
|  | BoLA-5:00301 | 47 | 55 | RIDGTHQTL | 1.1243 | Inside |
|  | BoLA-5:00301 | 40 | 48 | AGQASPSRI | 1.0592 | Inside |
|  | BoLA-5:00301 | 11 | 19 | AASLAAIAI | 0.7323 | Inside |
|  | BoLA-5:00301 | 23 | 31 | TANATVYMI | 0.6023 | Inside |
|  | BoLA-2:00501 | 22 | 30 | HTANATVYM | 0.5786 | Inside |
|  | BoLA-1:00901 | 5 | 13 | AQYAAQNPT | 0.5611 | Inside |
|  | BoLA-1:00901 | 22 | 30 | AQDPVATAA | 0.5385 | Inside |
|  | BoLA-5:00301 | 9 | 17 | AAAASLAAI | 0.524 | Inside |
|  | BoLA-2:00501 | 21 | 29 | MAQDPVATA | 0.5063 | Inside |
| MHC-II | HLA-DRB1*08:01 | 2 | 13 | INVQAKPAAAAS | 1.2401 | Inside |
|  | HLA-DRB1*08:01 | 3 | 14 | NVQAKPAAAASL | 1.2052 | Inside |
|  | HLA-DRB1*11:01 | 17 | 28 | IAIAFLAGCSST | 1.0748 | Outside |
|  | HLA-DRB1*04:01 | 106 | 117 | KLNPDVNLVDTL | 0.9319 | Outside |
|  | HLA-DRB1*11:01 | 110 | 121 | DVNLVDTLNGGE | 0.8499 | Outside |
|  | HLA-DRB1*03:01 | 31 | 42 | VSQDTSPKPATS | 0.8073 | Outside |
|  | HLA-DRB1*11:01 | 16 | 27 | AIAIAFLAGCSS | 0.7885 | Outside |
|  | HLA-DRB1*08:01 | 57 | 68 | ADLIGRGCAQYA | 0.7634 | Inside |
|  | HLA-DRB1*08:01 | 58 | 69 | DLIGRGCAQYAA | 0.7623 | Inside |
|  | HLA-DRB1*08:01 | 56 | 67 | AADLIGRGCAQY | 0.7607 | Inside |
|  | HLA-DRB1*11:01 | 15 | 26 | AAIAIAFLAGCS | 0.759 | Outside |
|  | HLA-DRB1*03:01 | 158 | 169 | VIAGQASPSRID | 0.7199 | Inside |
|  | HLA-DRB1*03:01 | 141 | 152 | DQLKTDAKLLSS | 0.6863 | Inside |
|  | HLA-DRB1*03:01 | 193 | 204 | NAGLVCGGVHTA | 0.6693 | Outside |
|  | HLA-DRB1*11:01 | 14 | 25 | LAAIAIAFLAGC | 0.6583 | Outside |
|  | HLA-DRB1*03:01 | 142 | 153 | QLKTDAKLLSSI | 0.6315 | Inside |
|  | HLA-DRB1*11:01 | 108 | 119 | NPDVNLVDTLNG | 0.6226 | Outside |
|  | HLA-DRB1*04:01 | 96 | 107 | LSTLTSALSGKL | 0.6116 | Outside |
|  | HLA-DRB1*11:01 | 188 | 199 | DLMVNNAGLVCG | 0.6107 | Outside |
|  | HLA-DRB1*03:01 | 194 | 205 | AGLVCGGVHTAN | 0.6107 | Outside |
|  | HLA-DRB1*03:01 | 81 | 92 | MAQDPVATAASN | 0.5404 | Inside |

**Table S4.** MHC-I and MHC-II epitopes of Peptidoglycan-binding protein ArfA.

| **Type** | **Allele** | **Start** | **End** | **Peptide** | **Antigenicity** | **Topology** |
| --- | --- | --- | --- | --- | --- | --- |
| MHC-I | BoLA-3:00401 | 313 | 321 | EGRAKNRRV | 2.4079 | Inside |
|  | BoLA-5:00301 | 85 | 93 | SRSGNTVTL | 2.0293 | Inside |
|  | BoLA-2:00501 | 315 | 323 | RAKNRRVEI | 1.5488 | Inside |
|  | BoLA-2:00501 | 36 | 44 | VVIPLLIAA | 1.5286 | Outside |
|  | BoLA-5:00301 | 265 | 273 | GSEGINIPL | 1.4901 | Outside |
|  | BoLA-3:00201 | 37 | 45 | VIPLLIAAI | 1.3697 | Outside |
|  | BoLA-3:00201 | 69 | 77 | TSTRGASAL | 1.368 | Inside |
|  | BoLA-3:00401 | 190 | 198 | NNIEVTGQA | 1.259 | Inside |
|  | BoLA-3:00201 | 73 | 81 | GASALSLSL | 1.2465 | Outside |
|  | BoLA-3:00201 | 145 | 153 | SVPIPDFGL | 1.1761 | Outside |
|  | BoLA-5:00301 | 34 | 42 | GAVVIPLLI | 1.1721 | Outside |
|  | BoLA-5:00301 | 76 | 84 | ALSLSLLSI | 1.1685 | Outside |
|  | BoLA-2:00501 | 290 | 298 | VAGDHIATV | 1.168 | Inside |
|  | BoLA-5:00301 | 152 | 160 | GLKVERDTV | 1.1611 | Inside |
|  | BoLA-5:00301 | 86 | 94 | RSGNTVTLI | 0.9596 | Inside |
|  | BoLA-3:00201 | 74 | 82 | ASALSLSLL | 0.9173 | Outside |
|  | BoLA-5:00301 | 83 | 91 | SISRSGNTV | 0.91 | Inside |
|  | BoLA-2:00501 | 143 | 151 | TASVPIPDF | 0.9009 | Outside |
|  | BoLA-3:00401 | 267 | 275 | EGINIPLSA | 0.8975 | Outside |
|  | BoLA-5:00301 | 154 | 162 | KVERDTVTL | 0.8428 | Inside |
|  | BoLA-5:00301 | 54 | 62 | QSVTGPTGV | 0.8273 | Outside |
|  | BoLA-3:00201 | 249 | 257 | ACPDARVTI | 0.8078 | Inside |
|  | BoLA-3:00201 | 138 | 146 | AEPVFTASV | 0.8049 | Outside |
|  | BoLA-2:00501 | 58 | 66 | GPTGVLPTL | 0.7673 | Outside |
|  | BoLA-3:00401 | 186 | 194 | MKIVNNIEV | 0.7437 | Inside |
|  | BoLA-1:00901 | 286 | 294 | VARGVAGDH | 0.7356 | Inside |
|  | BoLA-1:00901 | 163 | 171 | TGTAPSSEH | 0.6933 | Outside |
|  | BoLA-5:00301 | 55 | 63 | SVTGPTGVL | 0.6822 | Outside |
|  | BoLA-2:00501 | 136 | 144 | SSAEPVFTA | 0.6527 | Outside |
|  | BoLA-2:00801 | 217 | 225 | AVTGGPIAF | 0.6354 | Outside |
| MHC-II | HLA-DRB1*11:01 | 184 | 195 | PDMKIVNNIEVT | 1.3345 | Inside |
|  | HLA-DRB1*03:01 | 152 | 163 | GLKVERDTVTLT | 1.142 | Inside |
|  | HLA-DRB1*03:01 | 290 | 301 | VAGDHIATVGLG | 1.1015 | Outside |
|  | HLA-DRB1*03:01 | 289 | 300 | GVAGDHIATVGL | 1.0828 | Outside |
|  | HLA-DRB1*03:01 | 288 | 299 | RGVAGDHIATVG | 0.9966 | Inside |
|  | HLA-DRB1*11:01 | 35 | 46 | AVVIPLLIAAIG | 0.9839 | Outside |
|  | HLA-DRB1*03:01 | 31 | 42 | WLIGAVVIPLLI | 0.9588 | Outside |
|  | HLA-DRB1*11:01 | 36 | 47 | VVIPLLIAAIGY | 0.9571 | Outside |
|  | HLA-DRB1*03:01 | 124 | 135 | IHVDPVVRSLDF | 0.9458 | Outside |
|  | HLA-DRB1*11:01 | 34 | 45 | GAVVIPLLIAAI | 0.8752 | Outside |
|  | HLA-DRB1*03:01 | 37 | 48 | VIPLLIAAIGYG | 0.8578 | Outside |
|  | HLA-DRB1*03:01 | 74 | 85 | ASALSLSLLSIS | 0.8215 | Outside |
|  | HLA-DRB1*03:01 | 38 | 49 | IPLLIAAIGYGA | 0.8015 | Outside |
|  | HLA-DRB1*11:01 | 33 | 44 | IGAVVIPLLIAA | 0.7977 | Outside |
|  | HLA-DRB1*11:01 | 32 | 43 | LIGAVVIPLLIA | 0.7477 | Outside |
|  | HLA-DRB1*08:01 | 283 | 294 | DYLVARGVAGDH | 0.7378 | Outside |
|  | HLA-DRB1*03:01 | 153 | 164 | LKVERDTVTLTG | 0.7013 | Inside |
|  | HLA-DRB1*03:01 | 30 | 41 | PWLIGAVVIPLL | 0.6837 | Outside |
|  | HLA-DRB1*03:01 | 129 | 140 | VVRSLDFSSAEP | 0.6269 | Outside |
|  | HLA-DRB1*08:01 | 235 | 246 | ADYEILNRVADK | 0.5923 | Inside |
|  | HLA-DRB1*03:01 | 277 | 288 | RAKIVADYLVAR | 0.5736 | Inside |
|  | HLA-DRB1*04:01 | 302 | 313 | SVNPIASNATPE | 0.5279 | Inside |

**Table S5.** MHC-I and MHC-II epitopes of Chaperone protein DnaK.

| **Type** | **Allele** | **Start** | **End** | **Peptide** | **Antigenicity** | **Topology** |
| --- | --- | --- | --- | --- | --- | --- |
| MHC-I | BoLA-5:00301 | 528 | 536 | REAEGGSKV | 2.4434 | Inside |
|  | BoLA-3:00201 | 172 | 180 | GGGTFDVSL | 1.7383 | outside |
|  | BoLA-3:00201 | 247 | 255 | SSQSTSINL | 1.7265 | Inside |
|  | BoLA-5:00301 | 465 | 473 | KGTGKENTI | 1.7228 | Inside |
|  | BoLA-5:00301 | 191 | 199 | RATSGDNHL | 1.6117 | Inside |
|  | BoLA-5:00301 | 252 | 260 | SINLPYITV | 1.5051 | Inside |
|  | BoLA-3:00201 | 87 | 95 | TAPEISARI | 1.3212 | Inside |
|  | BoLA-5:00301 | 250 | 258 | STSINLPYI | 1.2953 | outside |
|  | BoLA-3:00401 | 31 | 39 | EGSRTTPSI | 1.2064 | Inside |
|  | BoLA-3:00201 | 91 | 99 | ISARILMKL | 1.1874 | Inside |
|  | BoLA-5:00301 | 365 | 373 | LLDVTPLSL | 1.0021 | outside |
|  | BoLA-1:00901 | 249 | 257 | QSTSINLPY | 0.9888 | outside |
|  | BoLA-5:00301 | 569 | 577 | KLGQESQAL | 0.8698 | Inside |
|  | BoLA-5:00301 | 533 | 541 | GSKVPEDTL | 0.8004 | outside |
|  | BoLA-2:00501 | 1 | 9 | MARAVGIDL | 0.7886 | Inside |
|  | BoLA-5:00301 | 363 | 371 | VLLLDVTPL | 0.7546 | outside |
|  | BoLA-5:00301 | 358 | 366 | GEVKDVLLL | 0.7261 | outside |
|  | BoLA-5:00301 | 34 | 42 | RTTPSIVAF | 0.653 | Inside |
|  | BoLA-3:00201 | 347 | 355 | VGAALQAGV | 0.6194 | outside |
| MHC-II | HLA-DRB1*08:01 | 459 | 470 | HVTAKDKGTGKE | 2.041 | Inside |
|  | HLA-DRB1*08:01 | 460 | 471 | VTAKDKGTGKEN | 1.8259 | Inside |
|  | HLA-DRB1*08:01 | 458 | 469 | VHVTAKDKGTGK | 1.6634 | Inside |
|  | HLA-DRB1*04:01 | 24 | 35 | PVVVANSEGSRT | 1.5284 | Outside |
|  | HLA-DRB1*04:01 | 25 | 36 | VVVANSEGSRTT | 1.5072 | Inside |
|  | HLA-DRB1*04:01 | 23 | 34 | DPVVVANSEGSR | 1.3484 | Outside |
|  | HLA-DRB1*03:01 | 614 | 625 | DAEVVDDGREAK | 1.2115 | Inside |
|  | HLA-DRB1*04:01 | 6 | 17 | GIDLGTTNSVVS | 1.1834 | Outside |
|  | HLA-DRB1*03:01 | 166 | 177 | ILVFDLGGGTFD | 1.0637 | Outside |
|  | HLA-DRB1*03:01 | 362 | 373 | DVLLLDVTPLSL | 0.9816 | Outside |
|  | HLA-DRB1*04:01 | 7 | 18 | IDLGTTNSVVSV | 0.8476 | Outside |
|  | HLA-DRB1*03:01 | 364 | 375 | LLLDVTPLSLGI | 0.8065 | Outside |
|  | HLA-DRB1*03:01 | 164 | 175 | QRILVFDLGGGT | 0.8004 | Outside |
|  | HLA-DRB1*03:01 | 363 | 374 | VLLLDVTPLSLG | 0.7228 | Outside |
|  | HLA-DRB1*04:01 | 325 | 336 | LVKELTGGKEPN | 0.7212 | Outside |
|  | HLA-DRB1*11:01 | 133 | 144 | AGQIAGLNVLRI | 0.6878 | Outside |
|  | HLA-DRB1*03:01 | 165 | 176 | RILVFDLGGGTF | 0.6516 | Outside |
|  | HLA-DRB1*03:01 | 266 | 277 | PLFLDEQLTRAE | 0.5874 | Outside |

**Table S6.** MHC-I and MHC-II epitopes of Protein GrpE.

| **Type** | **Allele** | **Start** | **End** | **Peptide** | **Antigenicity** | **Topology** |
| --- | --- | --- | --- | --- | --- | --- |
| MHC-I | BoLA-3:00401 | 10 | 18 | GNSGEQVTV | 1.9971 | Outside |
|  | BoLA-5:00301 | 156 | 164 | GGQGSKPVI | 1.9407 | Outside |
|  | BoLA-5:00301 | 23 | 31 | RIDPETGEV | 1.7826 | Inside |
|  | BoLA-2:00501 | 198 | 206 | ESVDDGTAV | 0.9464 | Outside |
|  | BoLA-2:00501 | 199 | 207 | SVDDGTAVA | 0.8491 | Outside |
|  | BoLA-5:00301 | 76 | 84 | ALRDQQAAA | 0.7676 | Inside |
|  | BoLA-3:00401 | 124 | 132 | DSALTGLGL | 0.716 | Outside |
|  | BoLA-2:00501 | 59 | 67 | TADLQRVQA | 0.6623 | Inside |
|  | BoLA-5:00301 | 75 | 83 | RALRDQQAA | 0.662 | Inside |
|  | BoLA-1:00901 | 65 | 73 | VQADFANYR | 0.6501 | Inside |
|  | BoLA-3:00201 | 138 | 146 | EGEDFDPVL | 0.6497 | Outside |
|  | BoLA-1:00901 | 86 | 94 | RAKASVVSQ | 0.6186 | Inside |
|  | BoLA-5:00301 | 83 | 91 | AADRAKASV | 0.6145 | Inside |
|  | BoLA-2:00601 | 141 | 149 | DFDPVLHEA | 0.5961 | Outside |
|  | BoLA-3:00401 | 112 | 120 | ESGPLKSVA | 0.5699 | Outside |
|  | BoLA-5:00301 | 54 | 62 | KVAELTADL | 0.5499 | Inside |
|  | BoLA-3:00201 | 170 | 178 | QGYQLGEQV | 0.5433 | Inside |
| MHC-II | HLA-DRB1*13:01 | 16 | 27 | VTVTDKRRIDPE | 1.2213 | Inside |
|  | HLA-DRB1*13:01 | 15 | 26 | QVTVTDKRRIDP | 1.063 | Inside |
|  | HLA-DRB1*13:01 | 14 | 25 | EQVTVTDKRRID | 0.9868 | Inside |
|  | HLA-DRB1*04:01 | 145 | 156 | VLHEAVQHEGDG | 0.9832 | Outside |
|  | HLA-DRB1*03:01 | 187 | 198 | VDTVVVDAAELE | 0.9045 | Outside |
|  | HLA-DRB1*13:01 | 13 | 24 | GEQVTVTDKRRI | 0.8843 | Inside |
|  | HLA-DRB1*03:01 | 188 | 199 | DTVVVDAAELES | 0.8153 | Outside |
|  | HLA-DRB1*03:01 | 190 | 201 | VVVDAAELESVD | 0.79 | Outside |
|  | HLA-DRB1*04:01 | 184 | 195 | VGVVDTVVVDAA | 0.66 | Outside |
|  | HLA-DRB1*13:01 | 129 | 140 | GLGLVAFGAEGE | 0.6529 | Outside |
|  | HLA-DRB1*08:01 | 71 | 82 | NYRKRALRDQQA | 0.5894 | Inside |
|  | HLA-DRB1*03:01 | 189 | 200 | TVVVDAAELESV | 0.5528 | Outside |
|  | HLA-DRB1*03:01 | 31 | 42 | VRHVPPGDMPGG | 0.5374 | Outside |
|  | HLA-DRB1*03:01 | 30 | 41 | EVRHVPPGDMPG | 0.536 | Outside |
|  | HLA-DRB1*04:01 | 186 | 197 | VVDTVVVDAAEL | 0.5237 | Outside |
|  | HLA-DRB1*03:01 | 28 | 39 | TGEVRHVPPGDM | 0.5231 | Outside |
|  | HLA-DRB1*04:01 | 183 | 194 | LVGVVDTVVVDA | 0.5098 | Outside |

**Table S7.** MHC-I and MHC-II epitopes of Lipoprotein LpqH.

| **Type** | **Allele** | **Start** | **End** | **Peptide** | **Antigenicity** | **Topology** |
| --- | --- | --- | --- | --- | --- | --- |
| MHC-I | BoLA-5:00301 | 27 | 35 | STTGSGETT | 3.1686 | Outside |
|  | BoLA-3:00401 | 55 | 63 | DGKDQNVTG | 3.1047 | Inside |
|  | BoLA-2:00501 | 29 | 37 | TGSGETTTA | 2.9268 | Inside |
|  | BoLA-5:00301 | 30 | 38 | GSGETTTAA | 2.6512 | Inside |
|  | BoLA-1:00901 | 117 | 125 | GQGNASATK | 2.3625 | Inside |
|  | BoLA-5:00301 | 115 | 123 | GTGQGNASA | 2.1536 | Outside |
|  | BoLA-5:00301 | 53 | 61 | VIDGKDQNV | 1.6321 | Inside |
|  | BoLA-1:00901 | 123 | 131 | ATKDGSHYK | 1.3982 | Inside |
|  | BoLA-1:00901 | 121 | 129 | ASATKDGSH | 1.2167 | Inside |
|  | BoLA-2:00501 | 89 | 97 | LTDGNPPEV | 1.191 | Outside |
|  | BoLA-5:00301 | 77 | 85 | AIGGAATGI | 1.1858 | Outside |
|  | BoLA-5:00301 | 46 | 54 | AASGPKVVI | 1.1397 | Inside |
|  | BoLA-3:00401 | 74 | 82 | VNIAIGGAA | 1.0858 | Inside |
|  | BoLA-2:00501 | 49 | 57 | GPKVVIDGK | 1.0762 | Inside |
|  | BoLA-3:00201 | 102 | 110 | LGNVNGVTL | 1.0494 | Outside |
|  | BoLA-5:00301 | 45 | 53 | GAASGPKVV | 1.0141 | Outside |
|  | BoLA-3:00201 | 48 | 56 | SGPKVVIDG | 0.9519 | Outside |
|  | BoLA-2:00501 | 80 | 88 | GAATGIAAV | 0.912 | Inside |
|  | BoLA-5:00301 | 8 | 16 | AVAGAAILV | 0.8775 | Outside |
|  | BoLA-2:00501 | 9 | 17 | VAGAAILVA | 0.7701 | Outside |
|  | BoLA-5:00301 | 6 | 14 | TVAVAGAAI | 0.7124 | Inside |
|  | BoLA-2:00501 | 62 | 70 | TGSVVCTTA | 0.6533 | Inside |
|  | BoLA-3:00201 | 81 | 89 | AATGIAAVL | 0.5918 | Inside |
| MHC-II | HLA-DRB1*04:01 | 19 | 30 | LSGCSSNKSTTG | 1.925 | Inside |
|  | HLA-DRB1*04:01 | 109 | 120 | TLGYTSGTGQGN | 1.9176 | outside |
|  | HLA-DRB1*04:01 | 110 | 121 | LGYTSGTGQGNA | 1.8231 | outside |
|  | HLA-DRB1*03:01 | 108 | 119 | VTLGYTSGTGQG | 1.797 | outside |
|  | HLA-DRB1*03:01 | 52 | 63 | VVIDGKDQNVTG | 1.6199 | Inside |
|  | HLA-DRB1*04:01 | 18 | 29 | GLSGCSSNKSTT | 1.5889 | outside |
|  | HLA-DRB1*03:01 | 107 | 118 | GVTLGYTSGTGQ | 1.5021 | outside |
|  | HLA-DRB1*03:01 | 106 | 117 | NGVTLGYTSGTG | 1.3731 | outside |
|  | HLA-DRB1*03:01 | 73 | 84 | NVNIAIGGAATG | 1.3399 | Inside |
|  | HLA-DRB1*04:01 | 130 | 141 | YKITGTATGVDM | 1.2358 | Inside |
|  | HLA-DRB1*03:01 | 74 | 85 | VNIAIGGAATGI | 1.1435 | Inside |
|  | HLA-DRB1*04:01 | 65 | 76 | VVCTTAAGNVNI | 1.1028 | Inside |
|  | HLA-DRB1*04:01 | 64 | 75 | SVVCTTAAGNVN | 1.0893 | Inside |
|  | HLA-DRB1*03:01 | 75 | 86 | NIAIGGAATGIA | 1.0534 | Inside |
|  | HLA-DRB1*11:01 | 71 | 82 | AGNVNIAIGGAA | 1.0494 | outside |
|  | HLA-DRB1*03:01 | 87 | 98 | AVLTDGNPPEVK | 0.9971 | outside |
|  | HLA-DRB1*04:01 | 63 | 74 | GSVVCTTAAGNV | 0.9849 | Inside |
|  | HLA-DRB1*03:01 | 60 | 71 | NVTGSVVCTTAA | 0.9697 | Inside |
|  | HLA-DRB1*03:01 | 100 | 111 | VGLGNVNGVTLG | 0.9566 | outside |
|  | HLA-DRB1*03:01 | 61 | 72 | VTGSVVCTTAAG | 0.9464 | Inside |
|  | HLA-DRB1*11:01 | 72 | 83 | GNVNIAIGGAAT | 0.9452 | outside |
|  | HLA-DRB1*11:01 | 77 | 88 | AIGGAATGIAAV | 0.9312 | outside |
|  | HLA-DRB1*11:01 | 78 | 89 | IGGAATGIAAVL | 0.9206 | outside |
|  | HLA-DRB1*03:01 | 59 | 70 | QNVTGSVVCTTA | 0.898 | Inside |
|  | HLA-DRB1*03:01 | 76 | 87 | IAIGGAATGIAA | 0.8728 | outside |
|  | HLA-DRB1*04:01 | 62 | 73 | TGSVVCTTAAGN | 0.8489 | Inside |
|  | HLA-DRB1*03:01 | 15 | 26 | LVAGLSGCSSNK | 0.8193 | outside |
|  | HLA-DRB1*11:01 | 9 | 20 | VAGAAILVAGLS | 0.6117 | outside |
|  | HLA-DRB1*03:01 | 86 | 97 | AAVLTDGNPPEV | 0.6079 | outside |
|  | HLA-DRB1*03:01 | 14 | 25 | ILVAGLSGCSSN | 0.6065 | outside |
|  | HLA-DRB1*11:01 | 8 | 19 | AVAGAAILVAGL | 0.5669 | outside |
|  | HLA-DRB1*11:01 | 6 | 17 | TVAVAGAAILVA | 0.5539 | outside |
|  | HLA-DRB1*03:01 | 13 | 24 | AILVAGLSGCSS | 0.5067 | outside |

**Table S8.** Secondary structure analysis of the vaccine candidates.

| **Vaccine** | **Beta sheet** | **Alpha Helix** | **Coil** |
| --- | --- | --- | --- |
| bTBV1 | 15.89% | 14.15% | 69.96% |
| bTBV2 | 9.32% | 30.03% | 60.65% |
| bTBV3 | 14.48% | 21.63% | 63.89% |

**Table S9.** Validation score (Ramachandran plot) for bTBV1, bTBV2, and bTBV3 construct.

| Vaccine construct | Ramachandran plot | | | |
| --- | --- | --- | --- | --- |
|  | Residues in most favored regions | Additional allowed regions | Generously allowed regions | Disallowed regions |
| bTBV1 | 83.9% | 13.6% | 0.8% | 1.7% |
| bTBV2 | 87.6% | 10.4% | 1.2% | 0.8% |
| bTBV3 | 88.7% | 9% | 0.5% | 1.9% |

**Table S10.** Protein disulfide engineering score of bTBV3 vaccine construct.

| **Res1 Chain** | **Res1 Seq #** | **Res1 AA** | **Res2 Chain** | **Res2 Seq #** | **Res2 AA** | **Chi3** | **Energy** | **Sum B-Factors** |
| --- | --- | --- | --- | --- | --- | --- | --- | --- |
| A | 1 | GLU | A | 4 | ALA | 106.18 | 3.95 | 0 |
| A | 53 | ALA | A | 57 | ALA | 108.01 | 4.65 | 0 |
| A | 102 | VAL | A | 105 | ALA | 100.02 | 4.99 | 0 |
| A | 111 | GLU | A | 114 | ALA | 86.18 | 1.34 | 0 |
| A | 129 | GLY | A | 176 | PRO | 88.48 | 1.45 | 0 |
| A | 141 | ALA | A | 144 | VAL | -93.67 | 5.59 | 0 |
| A | 142 | LYS | A | 193 | GLY | -66.16 | 2.69 | 0 |
| A | 143 | PHE | A | 191 | ALA | -73.78 | 3.86 | 0 |
| A | 145 | ALA | A | 189 | ILE | 98.57 | 7.65 | 0 |
| A | 147 | TRP | A | 186 | PRO | 85.81 | 2.9 | 0 |
| A | 155 | GLY | A | 176 | PRO | -69.43 | 2.67 | 0 |
| A | 164 | ALA | A | 171 | SER | -68.1 | 7.88 | 0 |
| A | 166 | MET | A | 169 | GLY | 90.8 | 2.83 | 0 |
| A | 181 | GLY | A | 237 | GLY | 101.58 | 3.12 | 0 |
| A | 200 | ALA | A | 222 | SER | 105.39 | 0.68 | 0 |
| A | 213 | THR | A | 301 | GLY | 116.11 | 4.03 | 0 |
| A | 214 | PHE | A | 245 | GLY | 84.29 | 5.3 | 0 |
| A | 217 | SER | A | 223 | SER | 98.75 | 2.4 | 0 |
| A | 233 | GLY | A | 319 | PRO | -100.33 | 2.67 | 0 |
| A | 234 | GLY | A | 258 | GLY | -100.3 | 5.01 | 0 |
| A | 234 | GLY | A | 275 | GLY | -64.08 | 3.36 | 0 |
| A | 247 | GLY | A | 263 | THR | 120.68 | 1.63 | 0 |
| A | 248 | SER | A | 264 | THR | 89.02 | 5.08 | 0 |
| A | 250 | SER | A | 262 | SER | -81.62 | 6.32 | 0 |
| A | 267 | GLY | A | 281 | ALA | 102.83 | 2.7 | 0 |
| A | 277 | GLY | A | 294 | VAL | -91.92 | 3.31 | 0 |
| A | 277 | GLY | A | 299 | THR | 113.3 | 2.65 | 0 |
| A | 278 | GLN | A | 299 | THR | 124.08 | 7.79 | 0 |
| A | 288 | GLY | A | 316 | GLY | 122.82 | 7.49 | 0 |
| A | 292 | PRO | A | 312 | THR | 84.72 | 2.91 | 0 |
| A | 292 | PRO | A | 342 | ALA | -114.47 | 4.66 | 0 |
| A | 297 | VAL | A | 335 | GLY | -80.84 | 4.81 | 0 |
| A | 303 | GLY | A | 306 | ASP | -111.56 | 4.59 | 0 |
| A | 310 | VAL | A | 329 | ALA | -101.77 | 2.15 | 0 |
| A | 320 | GLY | A | 323 | VAL | 102.02 | 5.27 | 0 |
| A | 324 | ALA | A | 351 | LEU | -99.94 | 3.53 | 0 |
| A | 325 | GLY | A | 352 | GLY | -105.9 | 3.15 | 0 |
| A | 331 | VAL | A | 335 | GLY | 106.2 | 2.36 | 0 |
| A | 337 | GLY | A | 340 | GLY | -84.6 | 3.22 | 0 |
| A | 350 | GLY | A | 353 | PRO | -83.54 | 4.87 | 0 |
| A | 357 | PRO | A | 395 | ALA | 88.44 | 5.59 | 0 |
| A | 358 | VAL | A | 376 | VAL | -107.84 | 5.52 | 0 |
| A | 379 | ALA | A | 386 | GLY | 117.96 | 1.9 | 0 |
| A | 387 | PRO | A | 447 | GLY | 67.03 | 6.68 | 0 |
| A | 388 | GLY | A | 411 | VAL | -87.9 | 4.75 | 0 |
| A | 389 | PRO | A | 444 | TYR | -90.71 | 5.43 | 0 |
| A | 389 | PRO | A | 446 | SER | -112.54 | 4.76 | 0 |
| A | 393 | HIS | A | 411 | VAL | 106.08 | 2.51 | 0 |
| A | 394 | GLU | A | 397 | GLN | 124.39 | 7.45 | 0 |
| A | 395 | ALA | A | 410 | THR | 117.24 | 4.32 | 0 |
| A | 396 | VAL | A | 405 | GLY | 96.89 | 3.55 | 0 |
| A | 414 | ASP | A | 417 | GLU | 113.48 | 2.57 | 0 |
| A | 422 | GLY | A | 449 | GLY | -67.9 | 4.19 | 0 |
| A | 428 | TYR | A | 443 | GLY | -114.88 | 7.23 | 0 |
| A | 431 | GLY | A | 456 | GLN | 86.71 | 2.08 | 0 |
| A | 432 | THR | A | 453 | ALA | 108.4 | 3.55 | 0 |
| A | 433 | GLY | A | 444 | TYR | -105.12 | 1.99 | 0 |
| A | 433 | GLY | A | 446 | SER | -84.18 | 2.88 | 0 |
| A | 434 | GLN | A | 443 | GLY | 69.12 | 7.57 | 0 |
| A | 435 | GLY | A | 455 | LYS | 82.05 | 3.66 | 0 |
| A | 437 | GLY | A | 495 | PRO | -69.41 | 2.49 | 0 |
| A | 439 | GLY | A | 496 | GLY | -100.14 | 2.79 | 0 |
| A | 459 | PRO | A | 488 | GLN | 123.14 | 3.56 | 0 |
| A | 480 | ALA | A | 484 | ALA | -116.73 | 3.55 | 0 |
| A | 505 | TRP | A | 590 | ALA | -90.19 | 3.02 | 0 |
| A | 514 | LYS | A | 527 | VAL | -108 | 4.75 | 0 |
| A | 517 | GLN | A | 581 | SER | 84.73 | 1.18 | 0 |
| A | 523 | SER | A | 580 | ALA | 98.11 | 4.17 | 0 |
| A | 525 | GLU | A | 580 | ALA | 106.1 | 6.12 | 0 |
| A | 532 | LYS | A | 545 | VAL | -64.55 | 5.59 | 0 |
| A | 550 | MET | A | 554 | LYS | 99.5 | 2.49 | 0 |
| A | 551 | PRO | A | 578 | GLY | 91.25 | 6.04 | 0 |
| A | 556 | SER | A | 559 | LYS | 118.77 | 4.47 | 0 |
| A | 558 | ASN | A | 567 | THR | -91.58 | 2.77 | 0 |
| A | 563 | GLY | A | 566 | GLU | 77.54 | 4.84 | 0 |
| A | 571 | ALA | A | 589 | ALA | -107.39 | 4.61 | 0 |

**Table S11.** A list of the discontinuous B-cell epitopes predicted by the ElliPro server.

| **No** | **Residues** | **No of residues** | **Score** |
| --- | --- | --- | --- |
| 1 | A:E1, A:A2, A:A3, A:A4, A:K5, A:M6, A:A7, A:K8, A:L9, A:S10, A:T11, A:D12, A:E13, A:L14, A:L15, A:D16, A:A17, A:F18, A:K19, A:E20, A:M21, A:T22, A:L23, A:L24, A:E25, A:L26, A:S27, A:D28, A:F29, A:V30, A:K31, A:K32, A:F33, A:E34, A:E35, A:T36, A:F37, A:V39, A:T40, A:A41, A:A42, A:A43, A:P44, A:V45, A:A46, A:V47, A:A48, A:A49, A:A50, A:G51, A:A52, A:A53, A:P54, A:A55, A:G56, A:A57, A:A58, A:V59, A:E60, A:A61, A:A62, A:E63, A:E64 | 63 | 0.822 |
| 2 | A:H500, A:M501, A:G502, A:S503, A:D504, A:W505, A:S506, A:I507, A:E508, A:I509, A:D510, A:G511, A:K512, A:K513, A:K514, A:K515, A:N516, A:Q517, A:K518, A:P519, A:D520, A:G521, A:N522, A:S523, A:G524, A:E525, A:Q526, A:V527, A:T528, A:V529, A:T530, A:D531, A:K532, A:R533, A:R534, A:I535, A:D536, A:P537, A:E538, A:T539, A:G540, A:E541, A:V542, A:R543, A:H544, A:V545, A:P546, A:P547, A:G548, A:D549, A:M550, A:P551, A:G552, A:G553, A:K554, A:K555, A:S556, A:S557, A:N558, A:K559, A:S560, A:T561, A:T562, A:G563, A:S564, A:G565, A:E566, A:T567, A:T568, A:T569, A:A570, A:A571, A:G572, A:T573, A:T574, A:A575, A:S576, A:P577, A:G578, A:A579, A:A580, A:S581, A:G582, A:K583, A:K584, A:A585, A:K586, A:F587, A:V588, A:A589, A:A590, A:W591, A:T592, A:L593, A:K594, A:A595, A:A596, A:A597, A:G598, A:G599, A:G600, A:S601 | 102 | 0.764 |
| 3 | A:G94, A:L95, A:K96, A:E97, A:A98, A:K99, A:D100, A:L101, A:V102, A:D103, A:G104, A:A105, A:P106, A:K107, A:P108, A:L109, A:L110, A:E111, A:K112, A:V113, A:A114, A:K115, A:E116, A:A117, A:A118, A:D119, A:E120, A:A121, A:K122, A:A123, A:K124, A:L125, A:A127, A:A128, A:G129, A:A130, A:T131, A:V132, A:A138, A:A139, A:A141, A:K142, A:F143, A:V144, A:A145, A:A146, A:W147, A:T148, A:L149, A:K150, A:A151, A:A153, A:G154, A:G155, A:G156, A:S157, A:T158, A:G159, A:P160, A:G161, A:S162, A:V163, A:A164, A:G165, A:M166, A:G167, A:G168, A:G169, A:S171, A:Q172, A:D173, A:T174, A:S175, A:P176, A:K177, A:P178, A:A179, A:G180, A:G181, A:G182, A:S183, A:V184, A:I185, A:P186, A:L188, A:I189, A:A190, A:A191, A:I192, A:G193, A:G194, A:G195, A:S196, A:G197, A:A198, A:S199, A:L218, A:G219, A:I231, A:G237, A:Q238, A:G239, A:S240, A:K241 | 104 | 0.645 |
| 4 | A:G474, A:L475, A:G476, A:Q477, A:T478, A:P479, A:A480, A:T481, A:T482, A:D483, A:A484, A:R485, A:R486 | 13 | 0.628 |
| 5 | A:P423, A:T432, A:G433, A:Q434, A:G435, A:N436, A:G437, A:P438, A:G439, A:P440, A:G441, A:L442, A:T445, A:T448, A:G449, A:Q450, A:G451, A:N452, A:A453, A:K454, A:K455, A:Q456, A:A457, A:S460, A:R461, A:T487, A:Q488, A:K489, A:F490, A:Y491, A:R492, A:G493, A:S494, A:P495, A:G496, A:K497, A:K498, A:R499 | 38 | 0.589 |
| 6 | A:E73, A:A74, A:A75, A:G76, A:D77, A:K78, A:I80 | 7 | 0.553 |
| 7 | A:P242, A:E249, A:S250, A:V251, A:D252, A:D253, A:G254, A:T255, A:A256, A:S261, A:S262, A:T263, A:G265, A:G267, A:E268, A:T269, A:T270, A:G271, A:G272, A:G284, A:P285 | 21 | 0.508 |
