# Supplementary material for: In Silico Driven Multi-Epitope Subunit Candidate Vaccine against Bovine Tuberculosis
Source: Transbound Emerg Dis. 2024 Sep 4;2024:5534041. doi: 10.1155/2024/5534041 (PMC12016833; doi:10.1155/2024/5534041)
Supplement: Supplementary 2 — Figure 1: secondary structure predictions of vaccine constructs. (a) bTBV1, (b) bTBV2, and (c) bTBV3. Figure 2: validation map of (a) bTBV1 and (b) bTBV2 in a Ramachandran plot. Figure 3: adaptiveness of vaccine constructs bTBV3 in E. coli strain K12. [file 5534041.f2.docx]

**
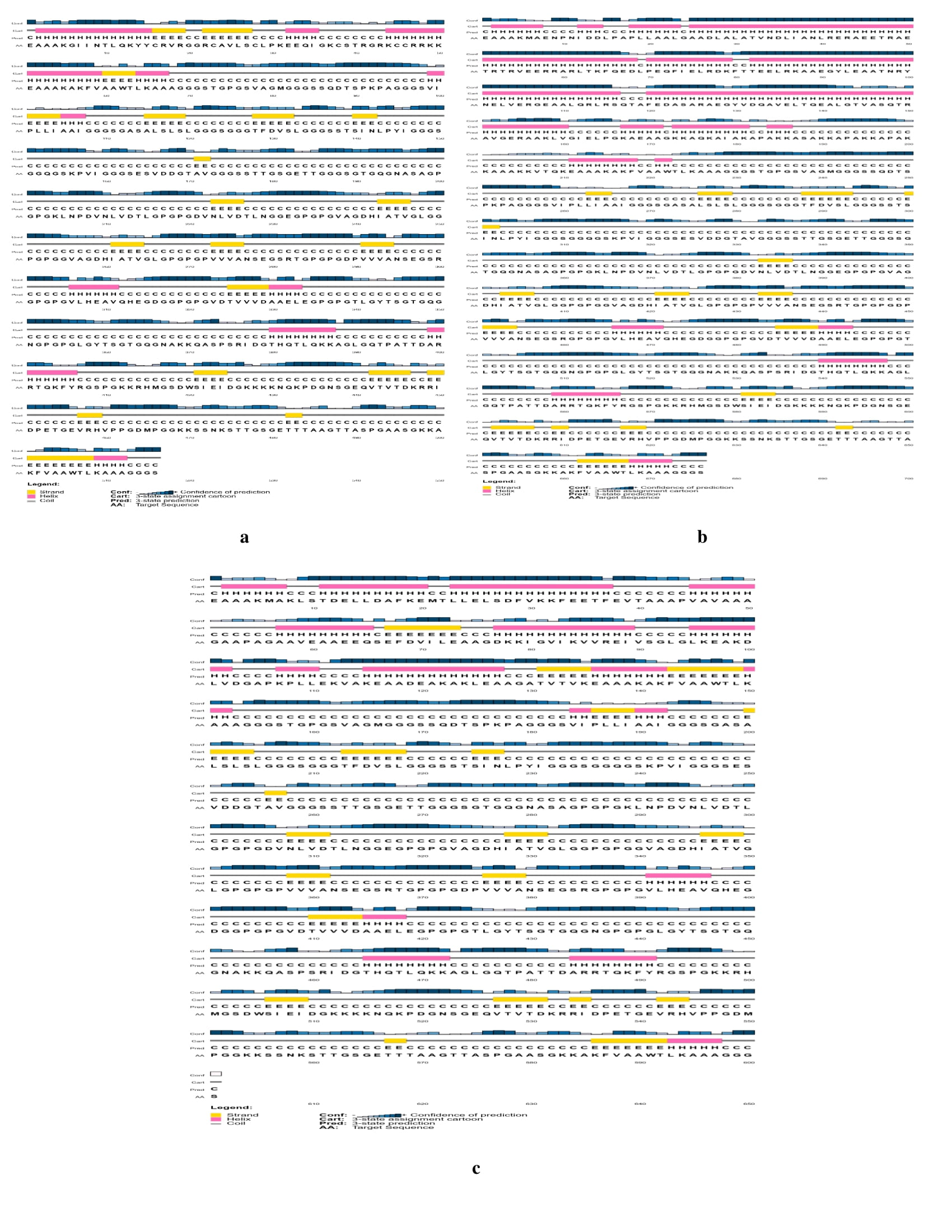
**

**Fig.S1. Secondary structure predictions of vaccine constructs.** (a) bTBV1, (b) bTBV2, and (c) bTBV3.


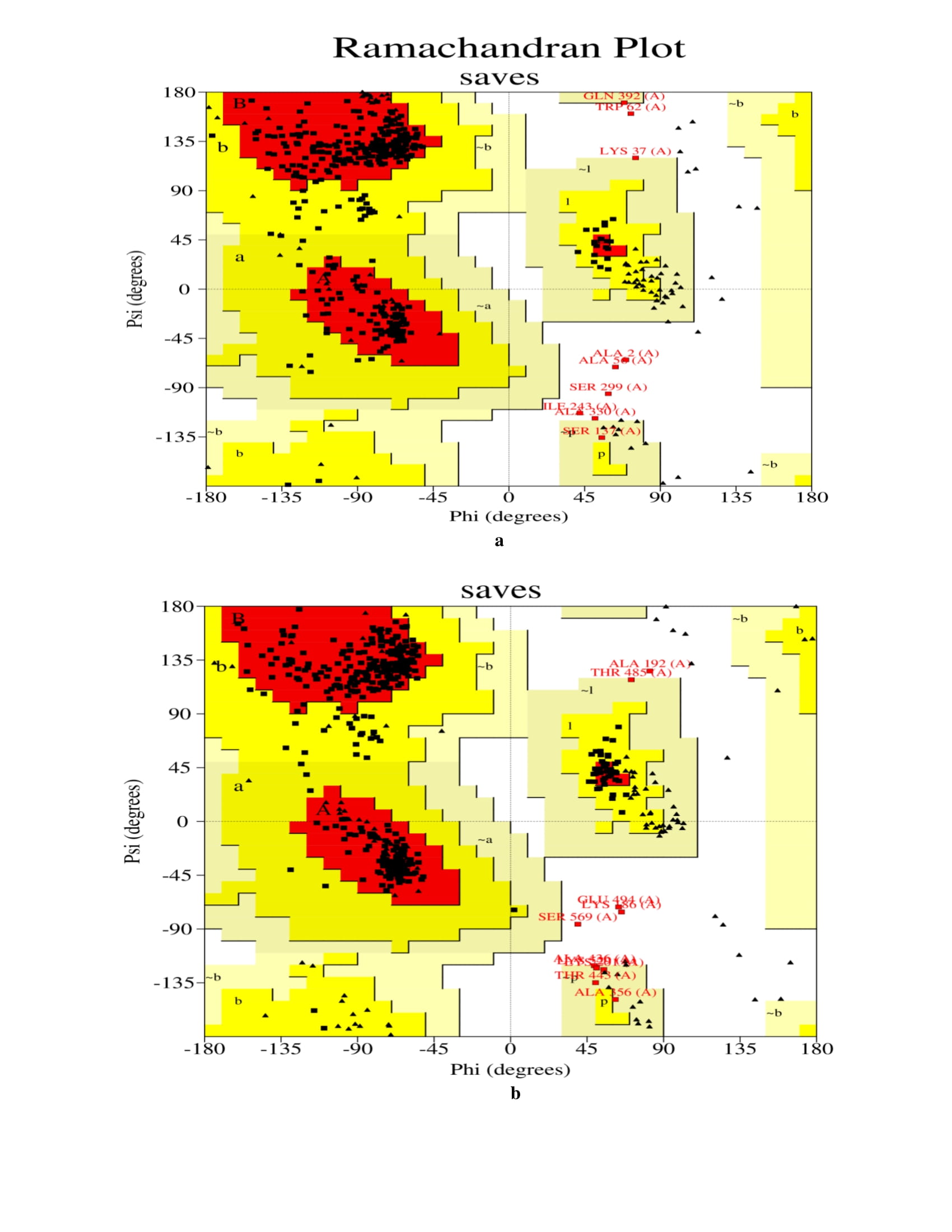


**Fig.S2.** Validation map of (a) bTBV1 and (b) bTBV2 in a Ramachandran plot.

**
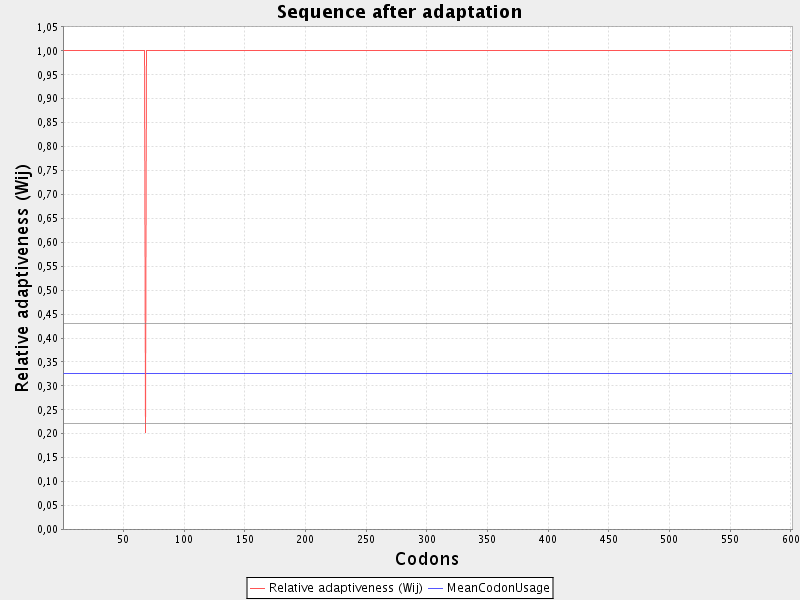
**

**Fig.S3.** Adaptiveness of vaccine construct bTBV3 in *E. coli* strain K12.
